# Harnessing redox properties of organic persistent radicals in SET-induced transformations

**DOI:** 10.3762/bjoc.22.102

**Published:** 2026-09-17

**Authors:** Jaysan Janabel, Tynchtyk Amatov

**Affiliations:** 1 Division of Science, New York University Abu Dhabi, Saadiyat Island, Abu Dhabi 129188, United Arab Emirates

**Keywords:** persistent radical effect, persistent radicals, redox catalysis, single-electron shuttle, single electron transfer

## Abstract

Organic persistent radicals are exceptionally versatile compounds known for their ability to undergo reversible oxidation and reduction. Their distinctive redox properties make them highly valuable in applications requiring reliable and predictable electron transfer. They exist in three stable redox states accessible through chemical or electrochemical oxidation and reduction processes. As such, organic persistent radicals can, in principle, serve as a platform for designing ground-state organic single-electron shuttle catalysts. This review highlights the unique chemistry of persistent radicals in mediating and promoting SET-induced transformations either in stoichiometric or catalytic settings. Particular emphasis is placed on persistent radical classes beyond nitroxides, such as TEMPO and its derivatives, which have historically dominated synthetic and catalytic applications.

## Introduction

The addition or removal of an electron is fundamental to many critical processes [1]. It is among the fundamental mechanisms driving a wide range of chemical transformations in biological and industrial processes, with significant applications in synthesis, energy storage, and environmental science. In the realm of synthetic organic chemistry, the importance of single-electron transfer (SET) has long been recognized in many ion-radical and radical-based reaction mechanisms [2]. The catalytic shuttling of an electron [3], a hallmark of redox catalysis and many electron transfer chain processes [4], has recently gained considerable momentum with the emergence of photoredox catalysis, which harnesses visible-light energy to selectively unlock the excited-state redox properties of molecular catalysts [5–7]. Mechanoredox catalysis is a more recent approach to redox catalysis enabled by piezoelectric materials that allow redox processes under mechanical stress such as ball milling or ultrasound [8–9].

Stable organic radicals (^P^R•) [10–12] are among the most versatile compounds capable of reversible oxidation and reduction [13]. In 1976, Griller and Ingold suggested that the term “persistent” should only refer to kinetic stability of radicals and “*be used to describe a radical that has a lifetime significantly greater than methyl under the same conditions*” while the term “stable” “*to describe a radical so persistent and so unreactive to air, moisture, etc., under ambient conditions that the pure radical can be handled and stored in the lab with no more precautions than would be used for the majority of commercially available organic chemicals*” [14]. Despite this distinction, the terms “persistent” and “stable” are often used interchangeably in the literature. While the two concepts are not synonymous, the distinction is not critical for the systems discussed herein, as all stable radicals considered in this review are also persistent.

**Figure 1:**
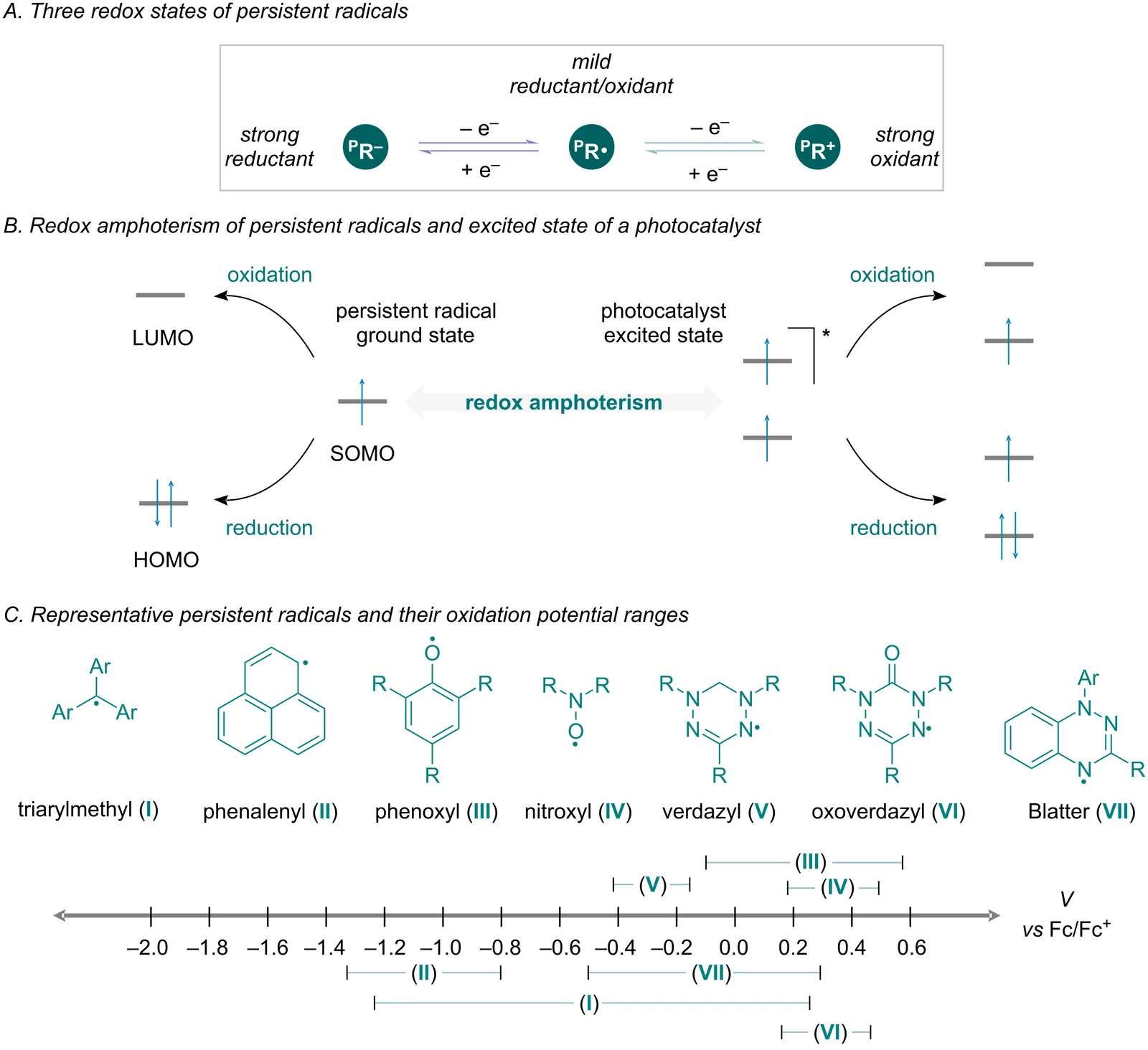
Three redox states of persistent radicals and oxidation potentials of different classes of persistent radicals.

Although organic persistent radicals have been known for several decades and their applications in organic synthesis have enabled extremely useful transformations, they have more recently emerged as a major focus of study in materials and biomaterials research, including batteries and sensors [15]. Their unique redox properties make them valuable in various applications requiring predictable, reversible electron transfer. They feature three stable redox states that can be accessed via chemical or electrochemical oxidation/reduction (Figure 1A). While persistent radicals are generally only mild reductants and oxidants, this property is often key to achieving selectivity in various transformations. Conversely, their one-electron-oxidized (^P^R^+^) or -reduced (^P^R^–^) forms exhibit stronger oxidizing and reducing properties, respectively. This review highlights how the redox interconversions of persistent radicals and their derivatives can lead to the development of innovative radical (functionalization) processes even in catalytic settings. Catalytic applications of persistent radicals in single electron transfer (SET) induced radical reactions are especially challenging, and might seem counterintuitive, as persistent radicals generally trap transient radicals with near diffusion-controlled kinetics.

Akin to the photocatalyst excited states, which can be quenched through either oxidative or reductive SET, the SOMO of radicals is also redox amphoteric (Figure 1B). As with excited states, the SOMO of persistent radicals can both donate and accept electrons. As such, organic persistent radicals could serve as a suitable platform for designing ground-state organic single-electron shuttle catalysts.

Not surprisingly, until recently, the catalytic applications of persistent radical redox chemistry have largely been limited to oxidative transformations that proceed via nonradical mechanisms. For example, the TEMPO/TEMPO^+^ redox pair has found numerous applications, mainly in functional-group redox conversions of alcohol/amine derivatives, as well as in other dehydrogenation reactions. In these reactions, TEMPO^+^ is the active oxidant that abstracts hydride from the substrate. Nitroxides [16–18], exemplified by TEMPO and its analogs, are among the most frequently utilized persistent radicals in synthetic organic chemistry. Other types of persistent radicals (Figure 1C), including carbon-centered triarylmethyl radicals (**I**) [19], oxygen-centered phenoxyl radicals (**III**) [20], nitrogen-centered radicals such as Kuhn verdazyls (**V**) and their oxo analogs (**VI**) [10,21–22] and Blatter radicals (**VII**) [23–24], are rarely employed in synthetically useful redox transformations. However, they could potentially facilitate reactions that are not achievable with nitroxides. Despite their stability rivaling that of nitroxide radicals, their applications in synthesis and catalysis remain unexplored and are only beginning to emerge. This contrasts with their easily tunable electrochemical properties, which span wide redox ranges and involve large cell potentials.

## Review

### The persistent radical effect and approaches to establishing the PRE-regime

**Figure 2:**
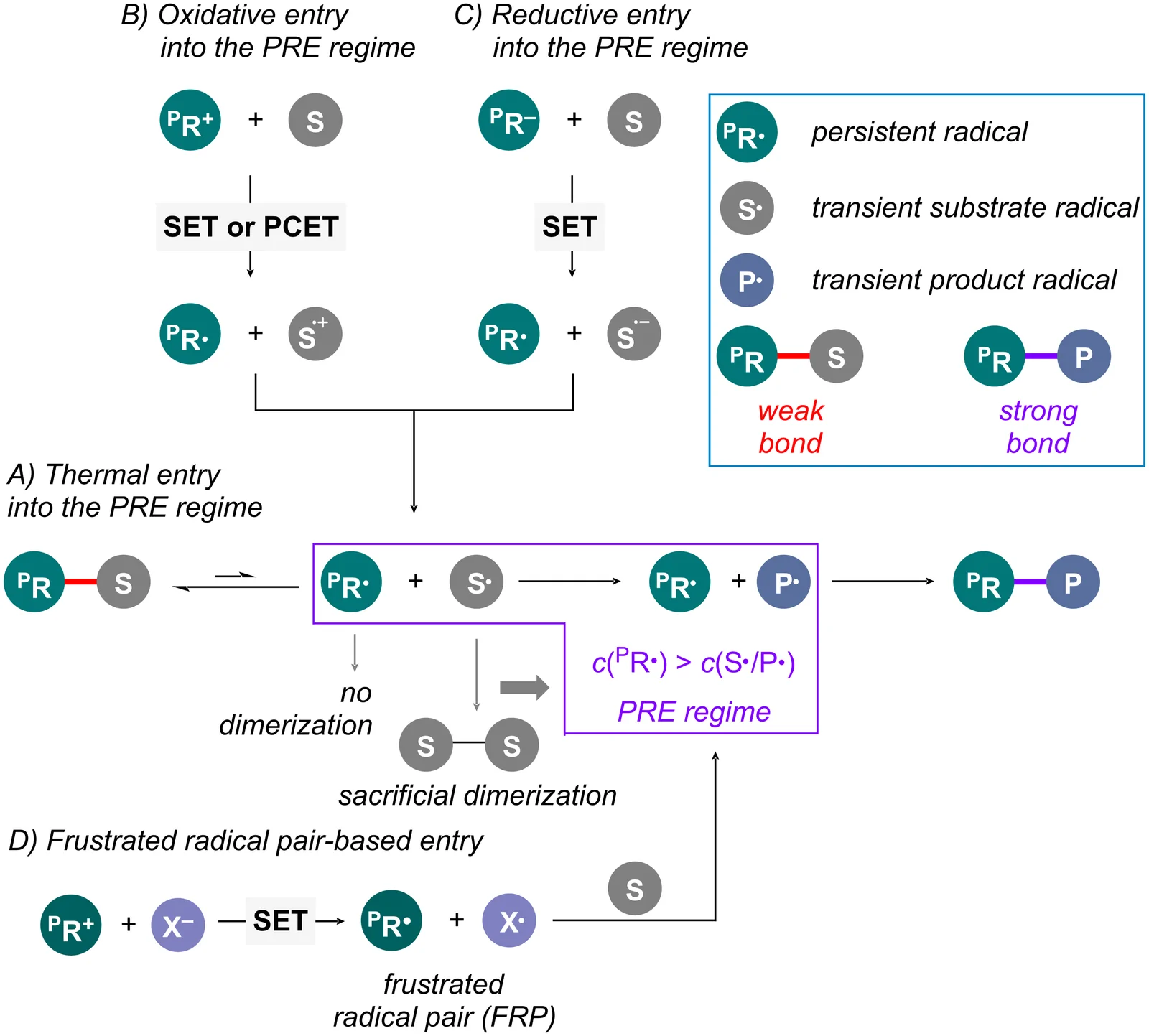
Approaches for accessing a PRE-regime.

TEMPO has long been used in mechanistic studies due to its fast kinetics in trapping transient carbon-centered radicals, thereby inhibiting radical processes. However, the same property of TEMPO can be harnessed to control the selectivity of radical reaction pathways and functionalization via radical trapping [25–27]. Perhaps the most distinctive and valuable property of persistent radicals is their ability to govern the selectivity of radical coupling processes enabled by the persistent radical effect (PRE) [28]. PRE is a kinetic regime that emerges when persistent and transient radicals are generated simultaneously, and the persistent radical accumulates while the transient radical concentration remains low (Figure 2). A salient feature of persistent radicals is their reluctance to undergo homocoupling, in contrast to transient radicals. As a result, even a small fraction of transient radical homocoupling produces a concentration imbalance, leading to a slight excess of persistent radicals over transient radicals. This concentration imbalance is central to the success of PRE, as even a minute excess of persistent radicals is typically enough to steer the reaction toward cross-coupling with transient radicals.

A common initiation step in a PRE-governed system involves the thermal or photochemical homolytic cleavage of a weak chemical bond, yielding a transient and a persistent radical in a 1:1 ratio (Figure 2A). Alternatively, a PRE-regime can be achieved by redox processes, either via SET-oxidation by ^P^R^+^ or SET-reduction by ^P^R^–^, respectively (Figure 2B and 2C). Recently, a new approach for entering the PRE-regime has emerged, based on the SET between redox-active ion pairs that generate frustrated radical pairs which evolve into PRE-governed systems (Figure 2D, vide infra).

**Scheme 1:**
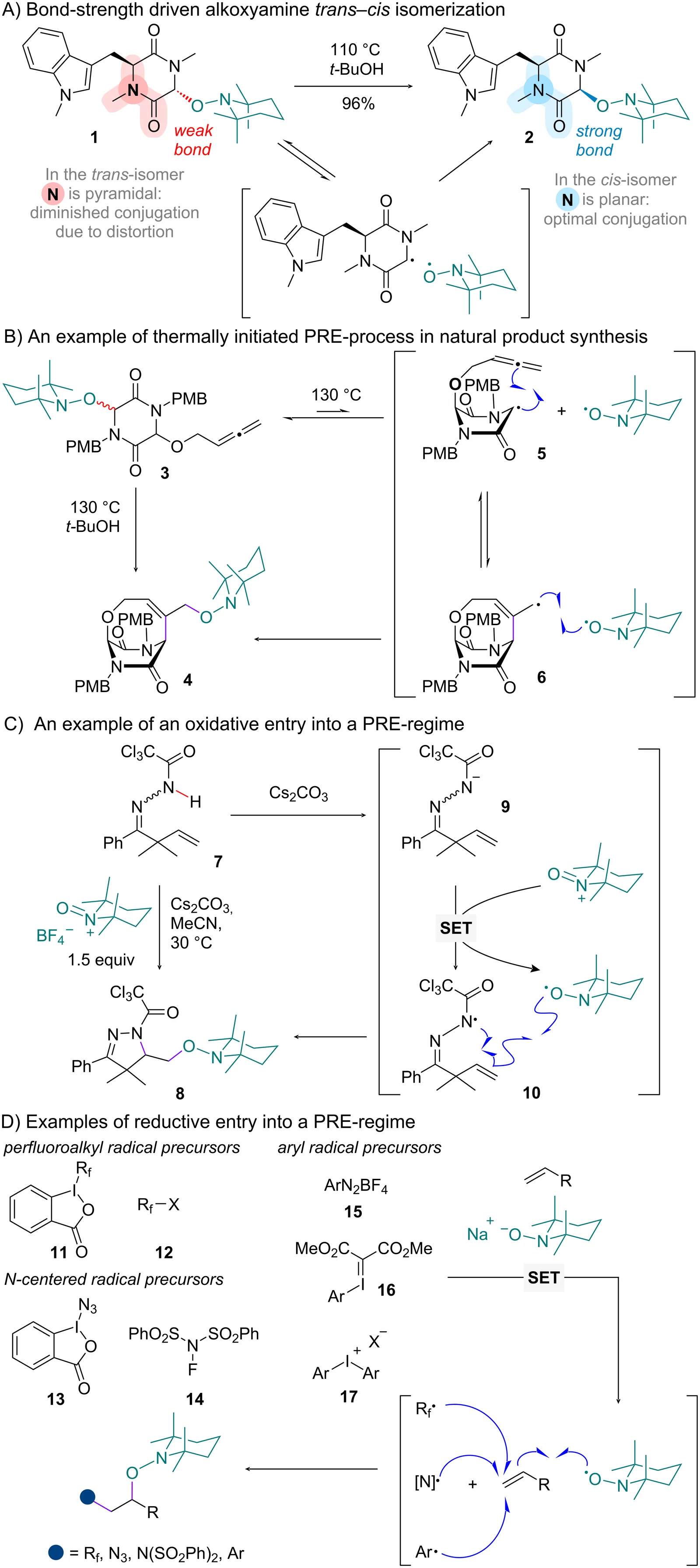
Representative examples of classical approaches to accessing the PRE-regime [34–35,38,40–45].

These types of reactivities, enabled by classical approaches to accessing the PRE regime (as depicted in Figure 2A–C) have been covered in several review articles [29–33], and only selected examples will be highlighted here to illustrate the concepts. Most of the reported synthetically useful PRE-governed processes that harness the single-electron-redox behavior of persistent radicals or their oxidized/reduced forms have been demonstrated using nitroxides such as TEMPO and their derivatives. Under conditions that lead to the formation of similar chemical bonds, differences in bond strength dictate selectivity. A very convincing example of this was reported for diketopiperazine-derived alkoxyamines, as shown in Scheme 1A [34]. Several *trans*-substituted alkoxyamines have been reported to quantitatively isomerize upon heating to *cis*-isomers. Computational and crystallographic studies revealed that due to a significant nitrogen pyramidalization, the C–O bond in the *cis*-isomers is weakened.

Jahn and co-workers applied diketopiperazine-derived alkoxyamines in the synthesis of bridged diketopiperazine (DKP) alkaloids [35–37]. For example, in their formal synthesis of the antibiotic bicyclomycin, a DKP-derived alkoxyamine with a pendant allene group underwent efficient cycloisomerization via radical cyclization initiated by thermal bond homolysis to generate the bridged DKP core of bicyclomycin (Scheme 1B).

Han and co-workers applied a SET-oxidation of deprotonated acylhydrazone derivatives by TEMPO^+^ to generate a nitrogen-centered radical that cyclized onto a pendant alkene, delivering heterocyclic products after TEMPO-trapping of the product radical (Scheme 1C) [38]. The same group also reported a similar reaction where *N*-hydrazyl radical is generated from hydrazone and TEMPO radical *at high temperature*, likely by hydrogen atom transfer (HAT) [39].

Metallic sodium reduces TEMPO to provide a sodium salt of deprotonated TEMPOH, i.e., TEMPONa. TEMPONa is capable of inducing a SET reduction of a variety of substrates, including arenediazonium salts, hypervalent iodine reagents such as diaryliodonium salts, and the Togni reagent and its analogs. The reductive power of TEMPONa was leveraged by Studer and coworkers in various difunctionalizations of alkenes using arenediazonium salts **15**, hypervalent iodine reagents **11**, **13**, **16**, **17**, perfluoroalkyl halides **12** and other radical sources (Scheme 1D) [40–45]

### Entry into the PRE-regimes via frustrated radical pairs

**Figure 3:**
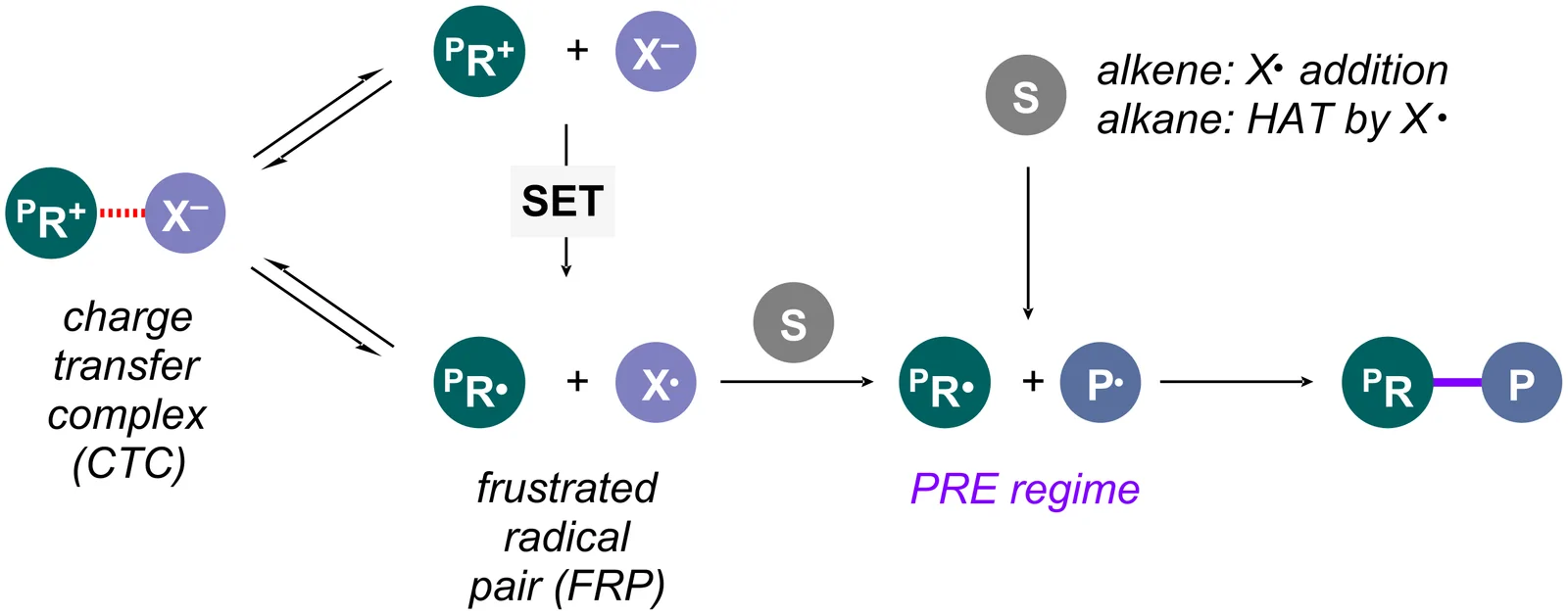
Evolution of FRPs into a PRE-regime.

Recently, conceptually distinct approaches to PRE-governed systems have emerged leveraging frustrated radical pairs (FRP) [46]. FRPs are radical pairs consisting of open-shell species that do not engage in homocoupling due to steric encumbrance and/or weak bonding interactions. Ionic FRPs are typically generated either by the SET process from frustrated Lewis pairs (FLPs) or by direct SET from ion-pairs/charge-transfer complexes (neutral FRPs) (Figure 3). In the latter case, the FRP usually consists of a persistent (^P^R•) and a transient heteroatom-centered radical (X•) which is capable of initiating radical transformations such as homolytic bond abstraction or addition to multiple bonds. Consequently, suitable FRP systems can evolve into a PRE-regime, allowing the unique chemistry of these systems to be leveraged to achieve challenging transformations.

**Scheme 2:**
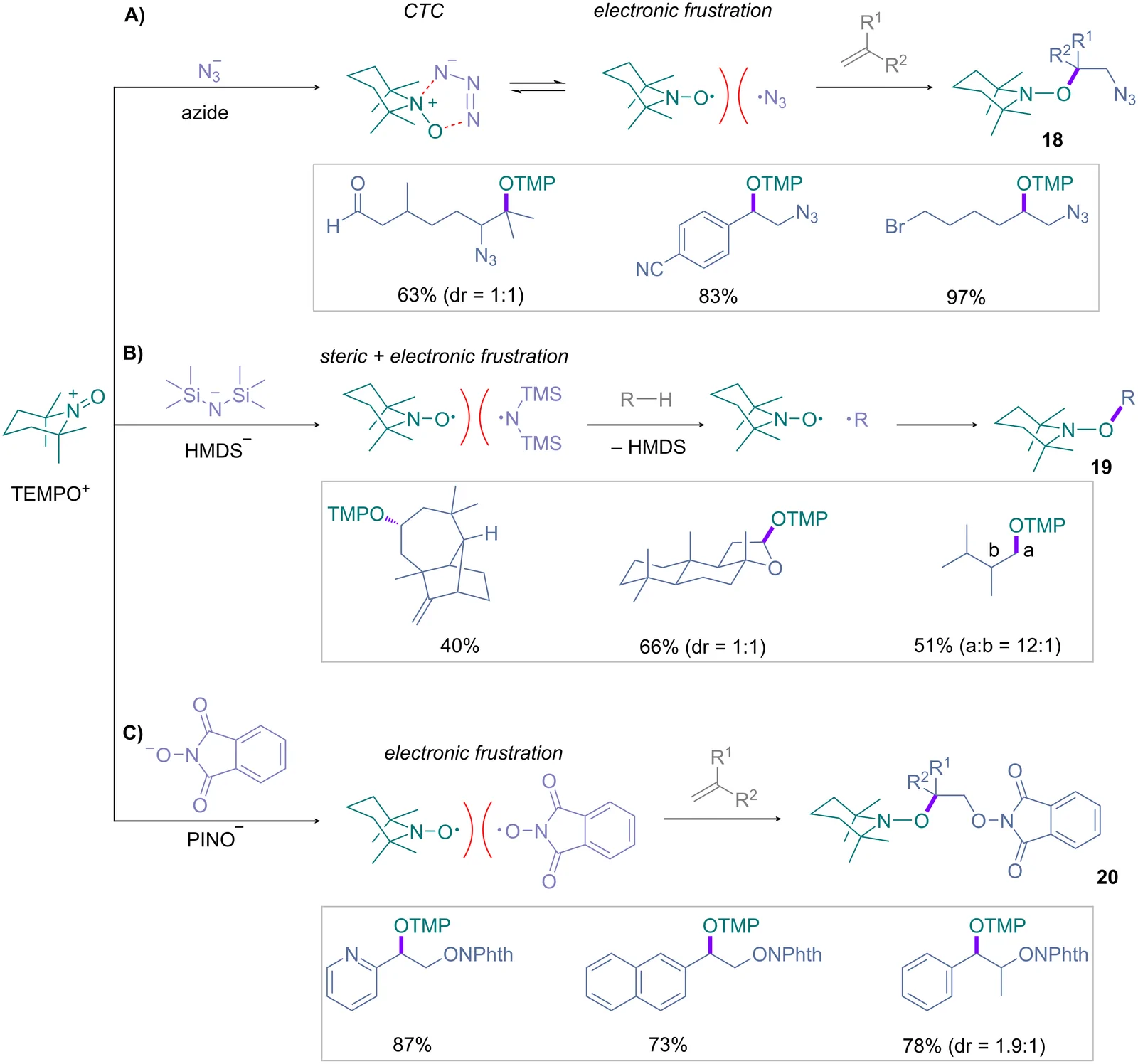
Examples of FRP in organic synthesis leveraging the redox properties of the TEMPO/TEMPO^+^ couple [47–50].

Recently, FRPs were put to use by the Lin group in olefin azidooxygenation [47] and challenging regioselective aliphatic C–H bond functionalization reactions (Scheme 2A and 2B, respectively) [48]. Chen and co-workers generated a TEMPO-PINO FRP system by the SET reduction of TEMPO^+^ with deprotonated *N*-hydroxyphthalimide (NHPI) which enabled the dioxygenation of alkenes (Scheme 2C) [49].

**Scheme 3:**
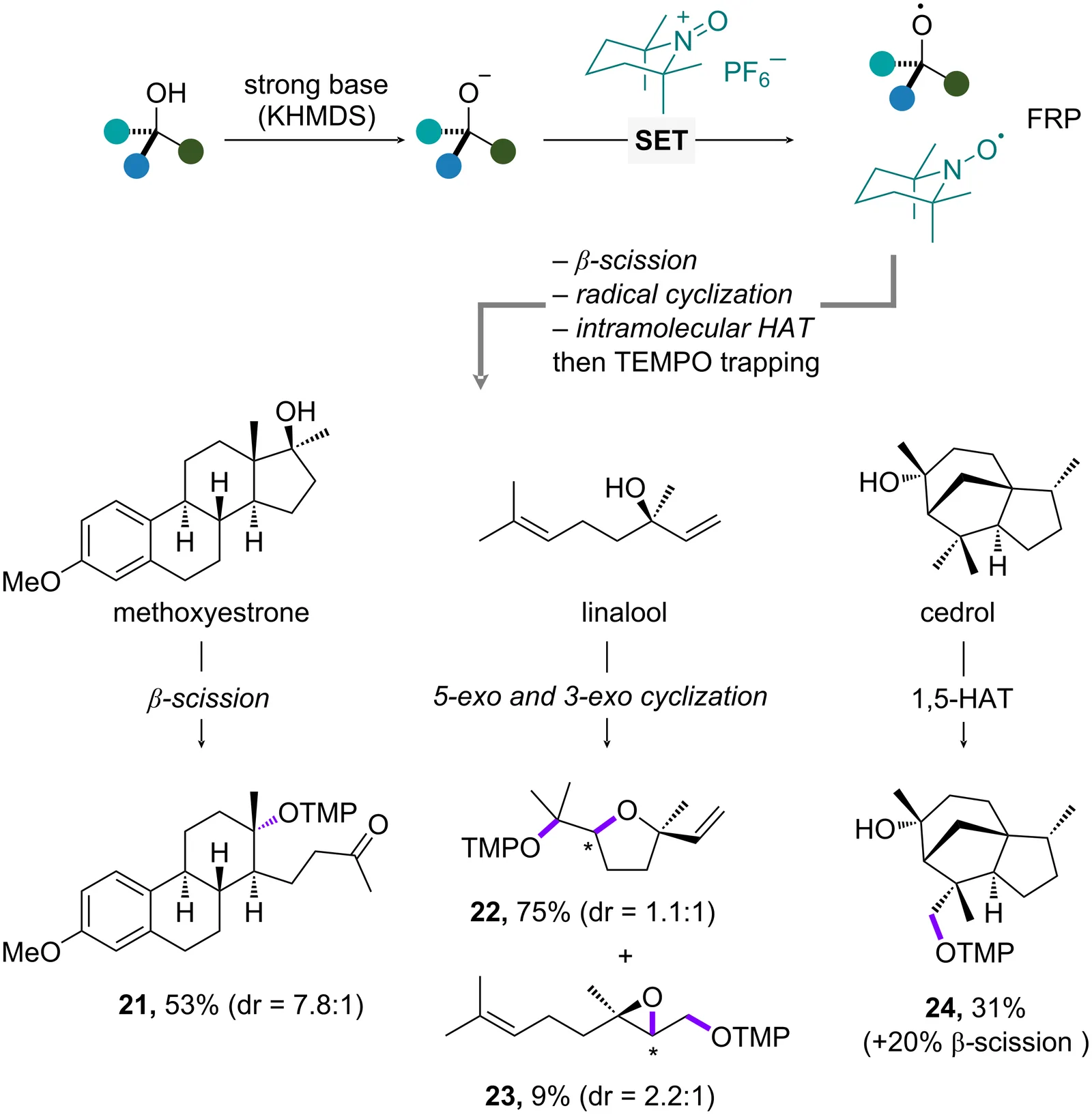
FRP formation via SET-oxidation of *tert*-alkoxides and applications [50].

Lin and co-workers also demonstrated that TEMPO^+^ can perform SET oxidation of tertiary alkoxides generated by deprotonation of tertiary alcohols (Scheme 3) [50]. The tertiary alkoxy radicals do not couple with TEMPO to generate stable species, and an FRP-like situation arises. Depending on the nature and structure of the tertiary alcohol, the corresponding alkoxy radicals can undergo C–C bond cleavage via β-scission. Alternatively, if a pendant double bond or alkyl C–H bond is present, the alkoxy radicals can undergo radical cyclization or intramolecular hydrogen atom abstraction (HAT) to generate carbon-centered radicals that can be trapped by TEMPO. The value of these transformations is especially apparent in the derivatization of natural products, such as steroids and terpenoids, that contain tertiary alcohol functional groups.

### Persistent radicals as SET oxidants

**Scheme 4:**
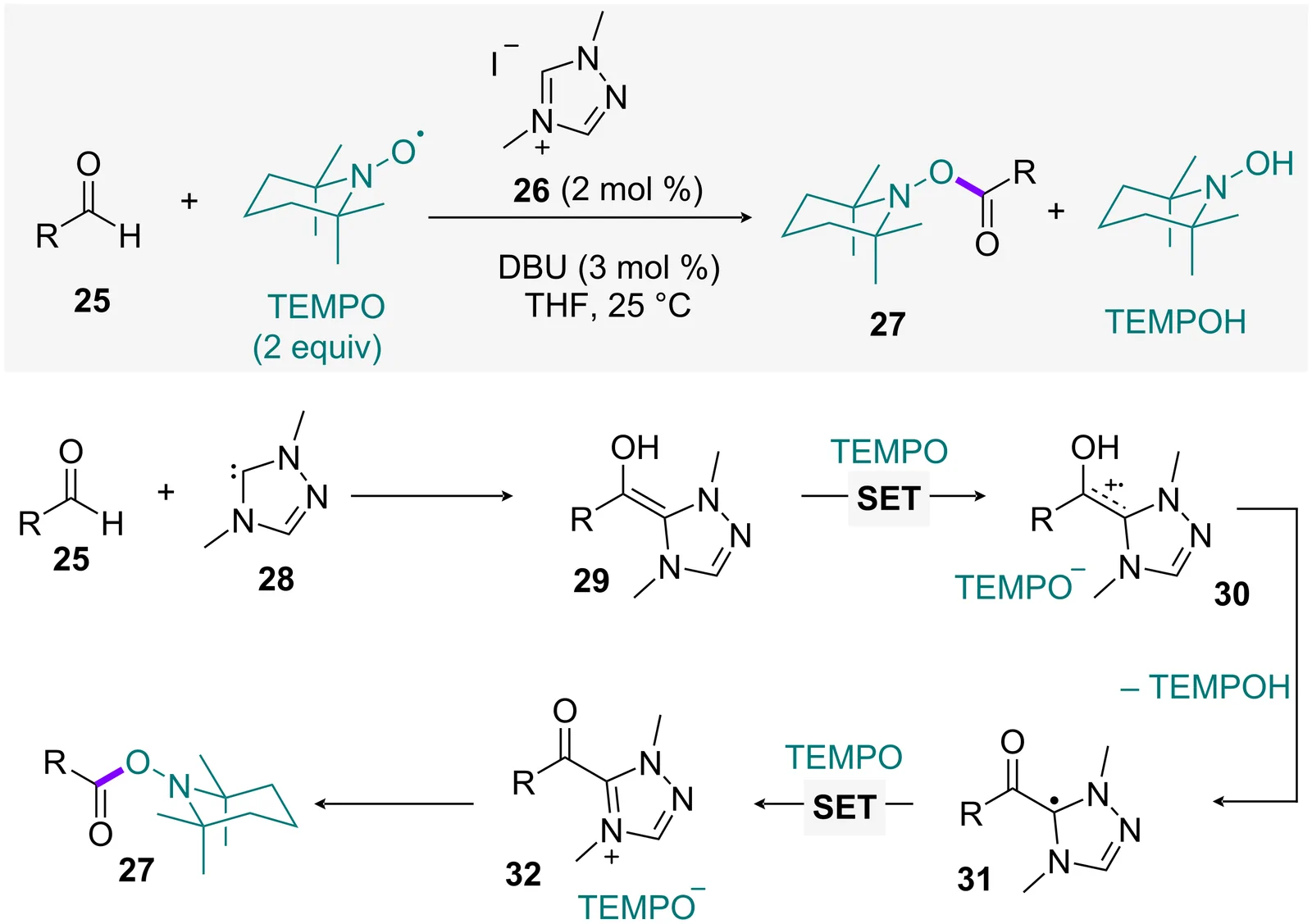
Biomimetic NHC-catalyzed aldehyde oxidation with TEMPO [51].

TEMPO in its neutral radical form can also act as a mild SET oxidant. In 2008, the Studer group disclosed an N-heterocyclic carbene (NHC)-catalyzed biomimetic oxidation of aldehydes with TEMPO as the terminal oxidant (Scheme 4) [51]. In this method, the consecutive double SET-oxidation of a Breslow intermediate **29** by TEMPO generates an acyl azolium/TEMPO^–^ ion pair **30** that rapidly collapses into TEMPO ester **27**, regenerating the NHC catalyst. The formation of TEMPO esters was so rapid that attempts to intercept the acyl azolium by other nucleophiles were unsuccessful. Later, an improvement by the Studer lab replaced TEMPO with a diphenoquinone-type organic oxidant, enabling the organocatalytic oxidative esterification of aldehydes [52]. These contributions by the Studer group also represent an early demonstration of the strong reducing power of Breslow intermediates, an area that has recently emerged as radical NHC catalysis [53].

**Scheme 5:**
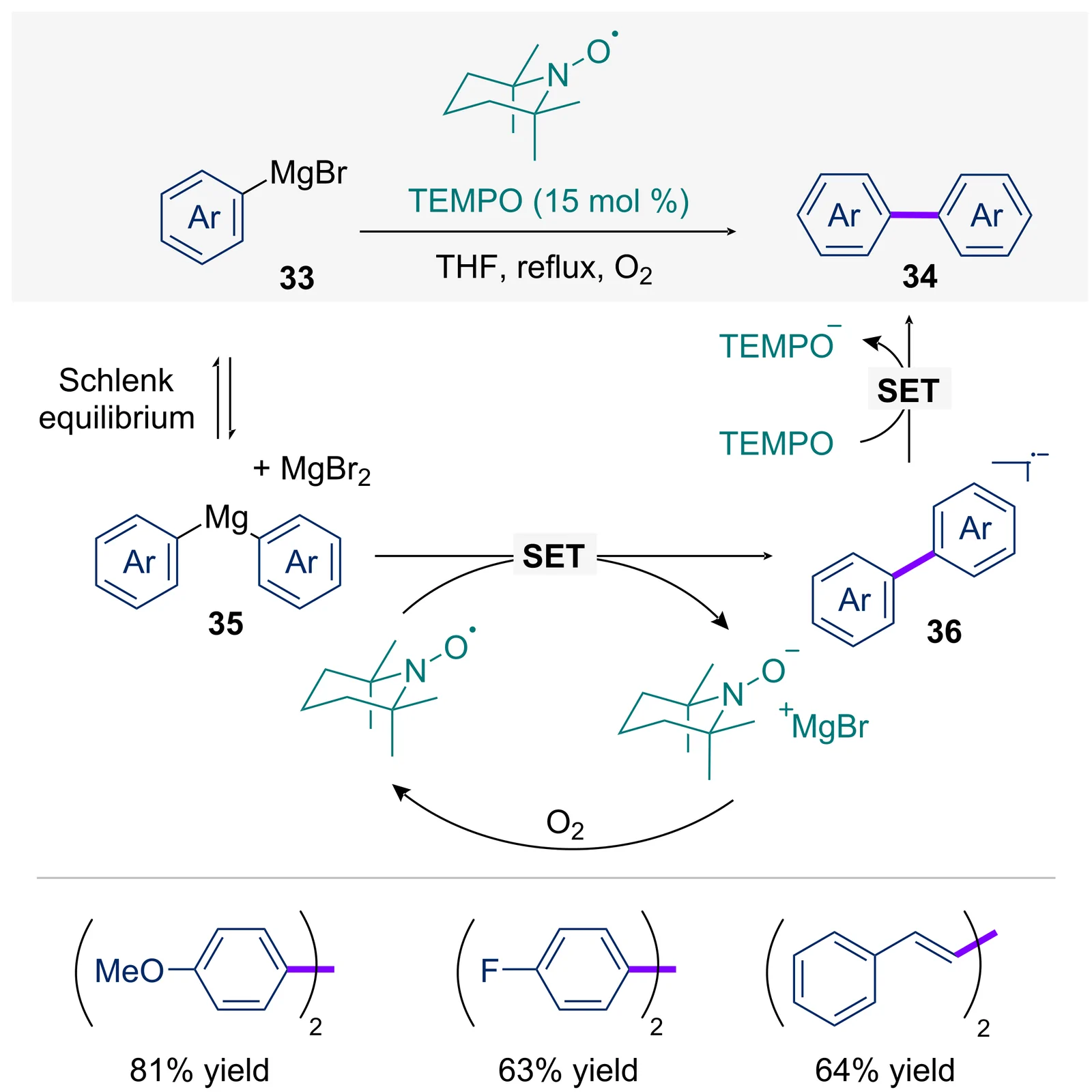
TEMPO-mediated oxidative homocoupling of Grignard reagents [58].

The oxidation of organolithium [54], alkylsamarium [55], and other organometallic compounds by TEMPO has been demonstrated in early works by Whitesides [54] and Curran [55], respectively. Studer and co-workers later showed in 2008 that a synthetically useful oxidative homocoupling of sp^2^ and sp-carbon centers from Grignard reagents is possible in the presence of two equivalents of TEMPO (Scheme 5) [56]. The TEMPO-mediated oxidative homocoupling of alkynyl- and alkenylmagnesium bromides and arylmagnesium bromides proceeded with similar efficiency. While the original proposal suggested a direct SET from Grignard reagents **33** to TEMPO, a subsequent mechanistic study revealed the important role of the Schlenk equilibrium and that diarylmagnesium species **35** are the SET reductants for TEMPO, activated via coordination to magnesium dibromide [57]. Indeed, the presence of MgBr_2_ was crucial for this reaction, and MgBr_2_-free diarylmagnesium compounds provided only traces of homocoupling products. Interestingly, it was demonstrated that TEMPO can be used in catalytic amounts through aerobic oxidation by purging with dry oxygen. While this approach only provides symmetric biaryls, in 2020 Studer and co-workers also developed a cross-coupling of biaryls via SET oxidation of in situ-generated mixed tetraarylborates with Bobbitt’s salt (4-acetylamino-2,2,6,6-tetramethyl-1-oxopiperidin-1-ium tetrafluoroborate) [58].

**Scheme 6:**
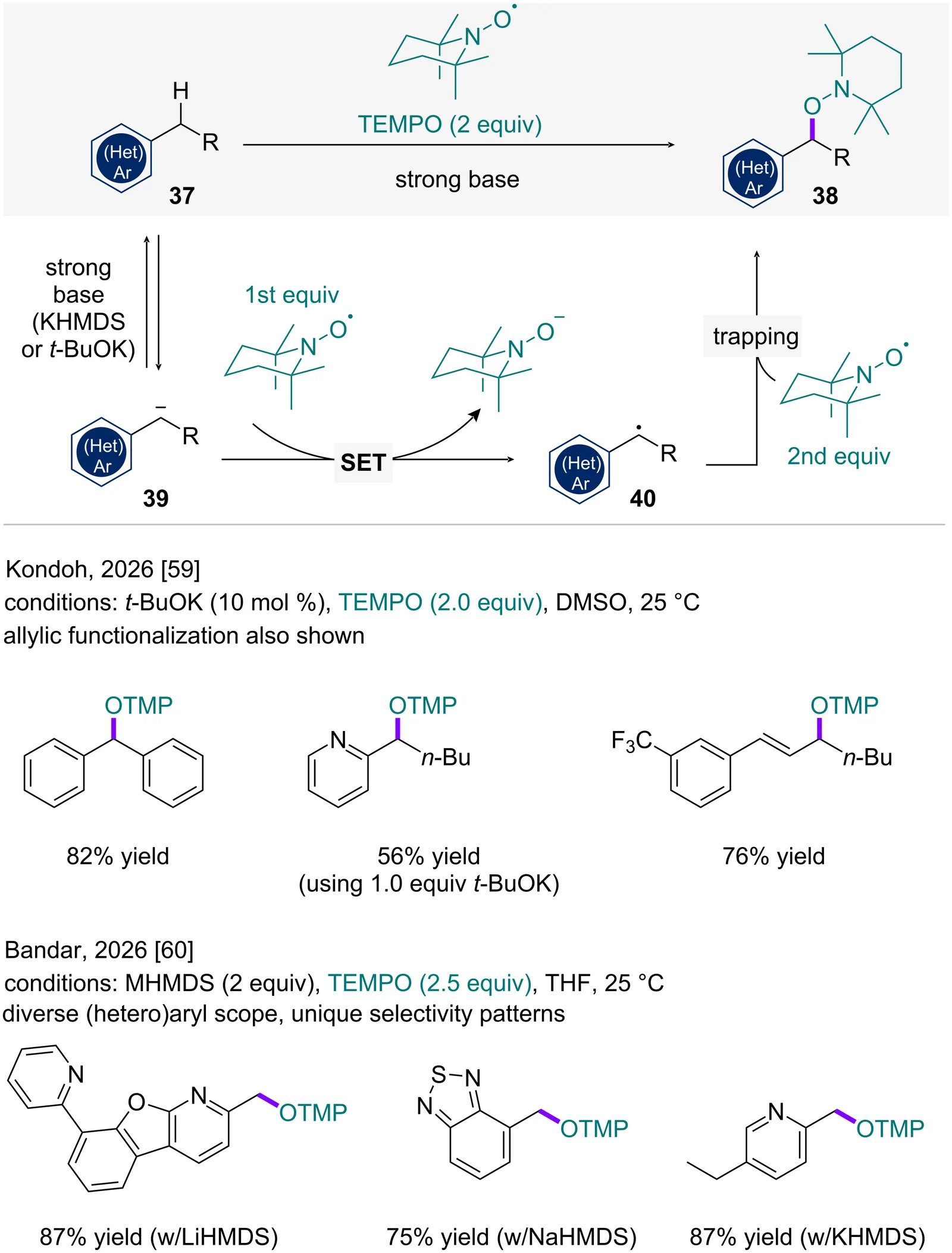
TEMPO-oxidation of benzylic carbanions for C–H aminoxylation [59–60].

Recently, the Kondoh [59] and Bandar [60] labs independently reported direct oxidative benzylic- and allylbenzylic-type C–H functionalizations (Scheme 6). Benzylic-type carbanions **39**, generated via deprotonation using strong bases such as potassium *tert-*butoxide (Kondoh) or alkali metal hexamethyldisilazides (Bandar), can be efficiently converted to benzylic alkoxyamines **38** in the presence of two equivalents of TEMPO. In these reactions, one equivalent of TEMPO acts as a SET oxidant to generate a benzylic radical **40** from the benzylic carbanion, and the second equivalent serves as a radical trap. Potential future directions can entail the use of *N*-centered verdazyl (**V**–**VI**) and Blatter (**VII**) radicals for similar aminative functionalizations.

### Persistent radicals as SET reductants

**Scheme 7:**
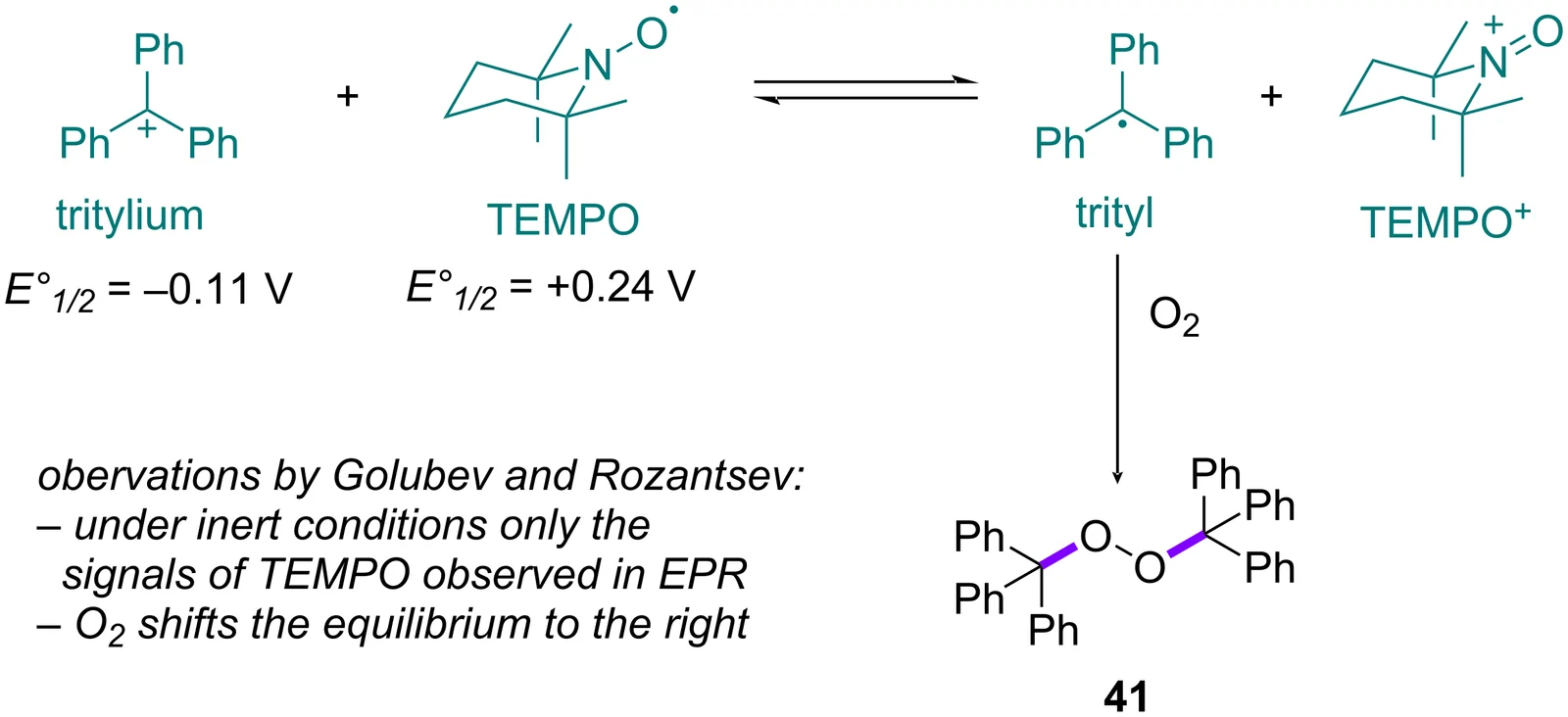
Shifting of thermodynamically uphill redox equilibrium between triphenylcarbenium and TEMPO in the presence of molecular oxygen [61].

Golubev, Rozantsev and co-workers reported in 1970 that TEMPO can reduce a trityl cation to a trityl radical [61]. This is a thermodynamically uphill SET process, and under inert conditions only the EPR signal corresponding to TEMPO is observed. However, in the presence of oxygen, the equilibrium shifts towards the oxidation of TEMPO, as the trityl radical reacts immediately with molecular oxygen to form peroxide **41** (Scheme 7).

**Scheme 8:**
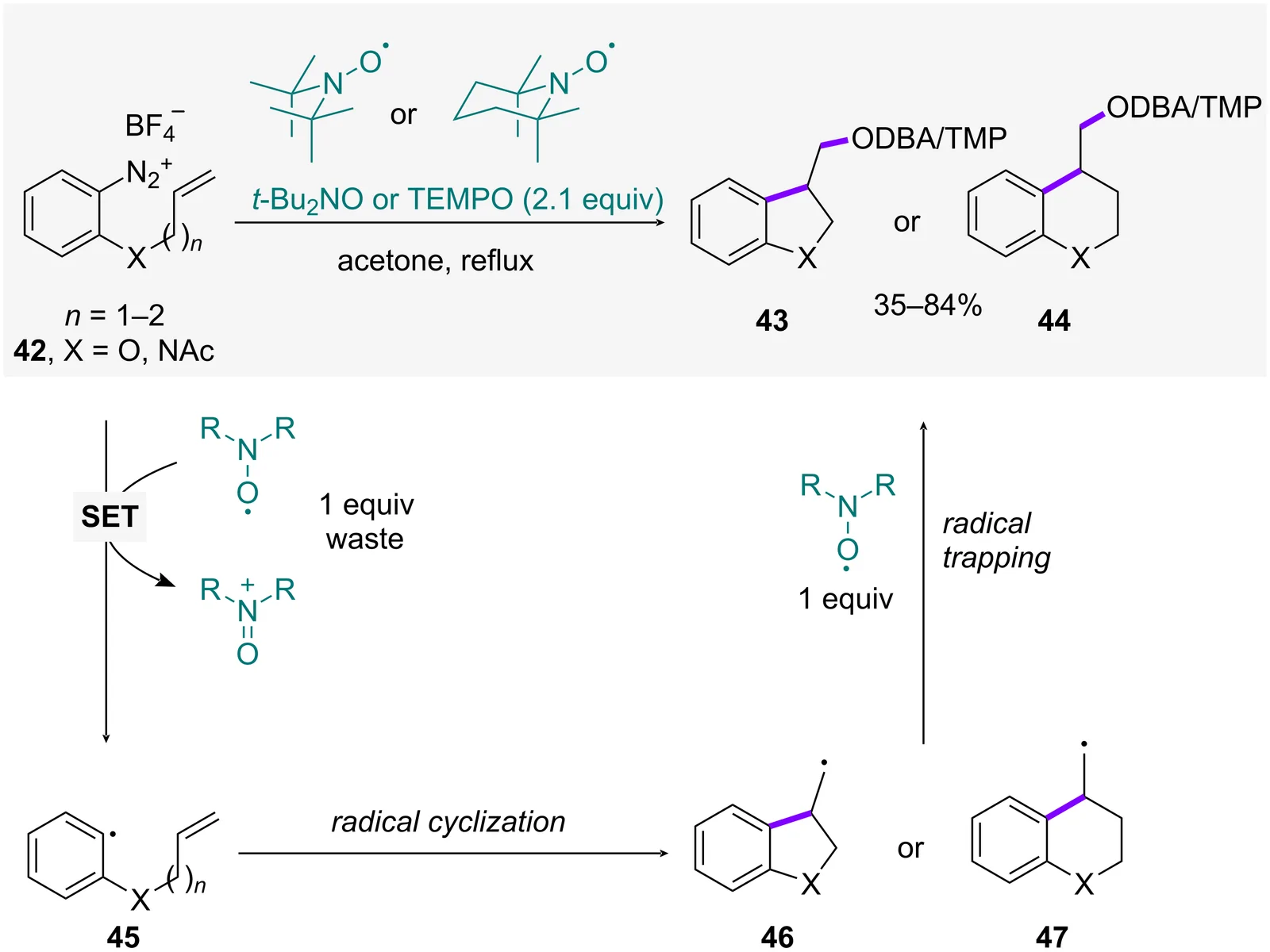
TEMPO or *t*-Bu_2_NO as a stoichiometric SET-reductant for the generation of aryl radicals from arenediazonium salts [62].

Arenediazonium salts were shown to be reduced by nitroxides such as di-*tert*-butylnitroxide (*t*-Bu_2_NO) and TEMPO under mild heating. This reaction was applied by Beckwith to the cyclization reactions of *o*-alkenyloxyarenediazonium salts **42** (Scheme 8) [62].

**Scheme 9:**
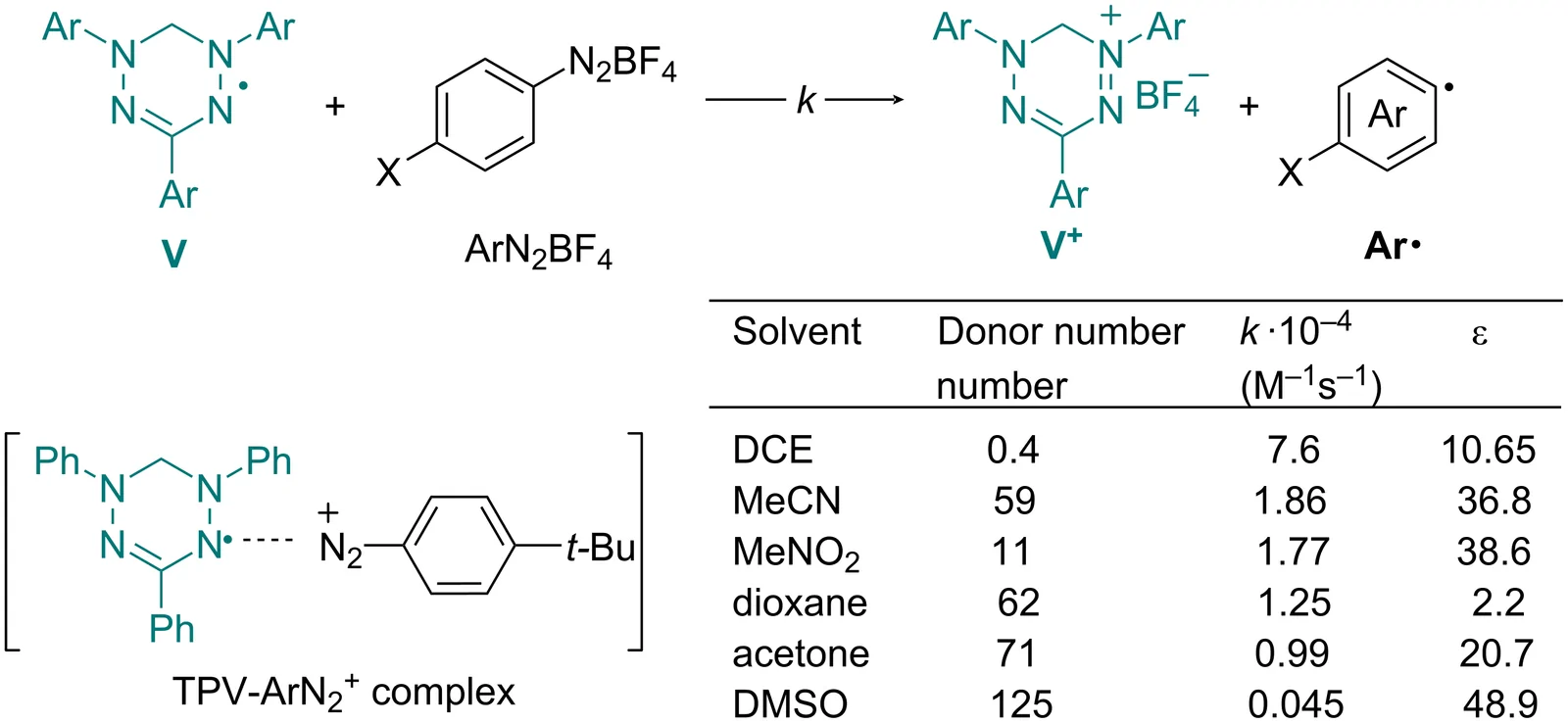
SET-reduction of arenediazonium salts by Kuhn verdazyls and rate constants of reduction of (4-*tert*-butylphenyl)diazonium tetrafluoroborate with triphenylverdazyl (TPV) [63].

In 1983, Bogillo, Gragerov, and co-workers reported that Kuhn-type verdazyls **V** react stoichiometrically with arenediazonium salts to generate aryl radicals (Scheme 9) [63]. They studied the kinetics of this reaction by UV–vis spectroscopy and determined a linear correlation between the redox potentials of arenediazonium salts and verdazyl radicals. Interestingly, a significant solvent dependence of reaction kinetics was observed, with the SET-reduction rate constant exhibiting a linear correlation with the solvent donor number [64], indicating specific solvation of the reagents in solution. This was interpreted as being more consistent with the formation of a donor–acceptor complex between the verdazyl radical and the arenediazonium salt.

### Heterointermediate reaction sequences and cooperative catalysis

**Figure 4:**
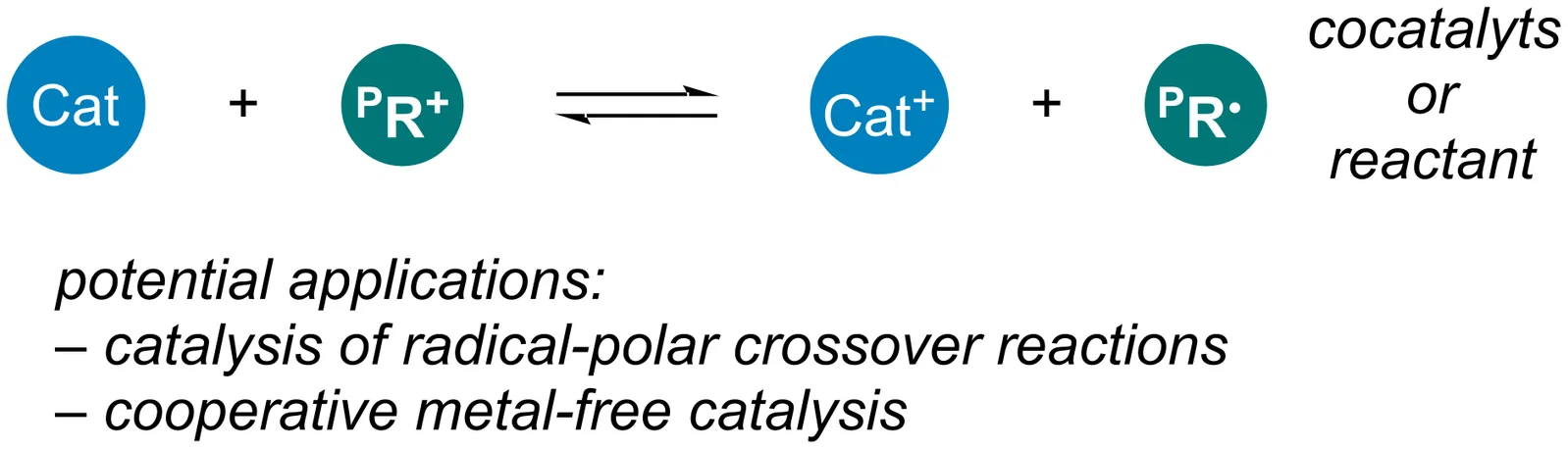
Reversible chemical redox process between persistent radical and redox-active cocatalysts.

The ability of persistent radicals to reversibly undergo single-electron redox chemistry with chemical oxidants or reductants enables their combination with other redox-active species in stoichiometric or co-catalytic settings to achieve attractive transformations. In such combinations, the persistent radical can act as a co-catalyst or a reactant, enabling more sustainable routes to catalytic or co-catalytic transformations (Figure 4).

**Scheme 10:**
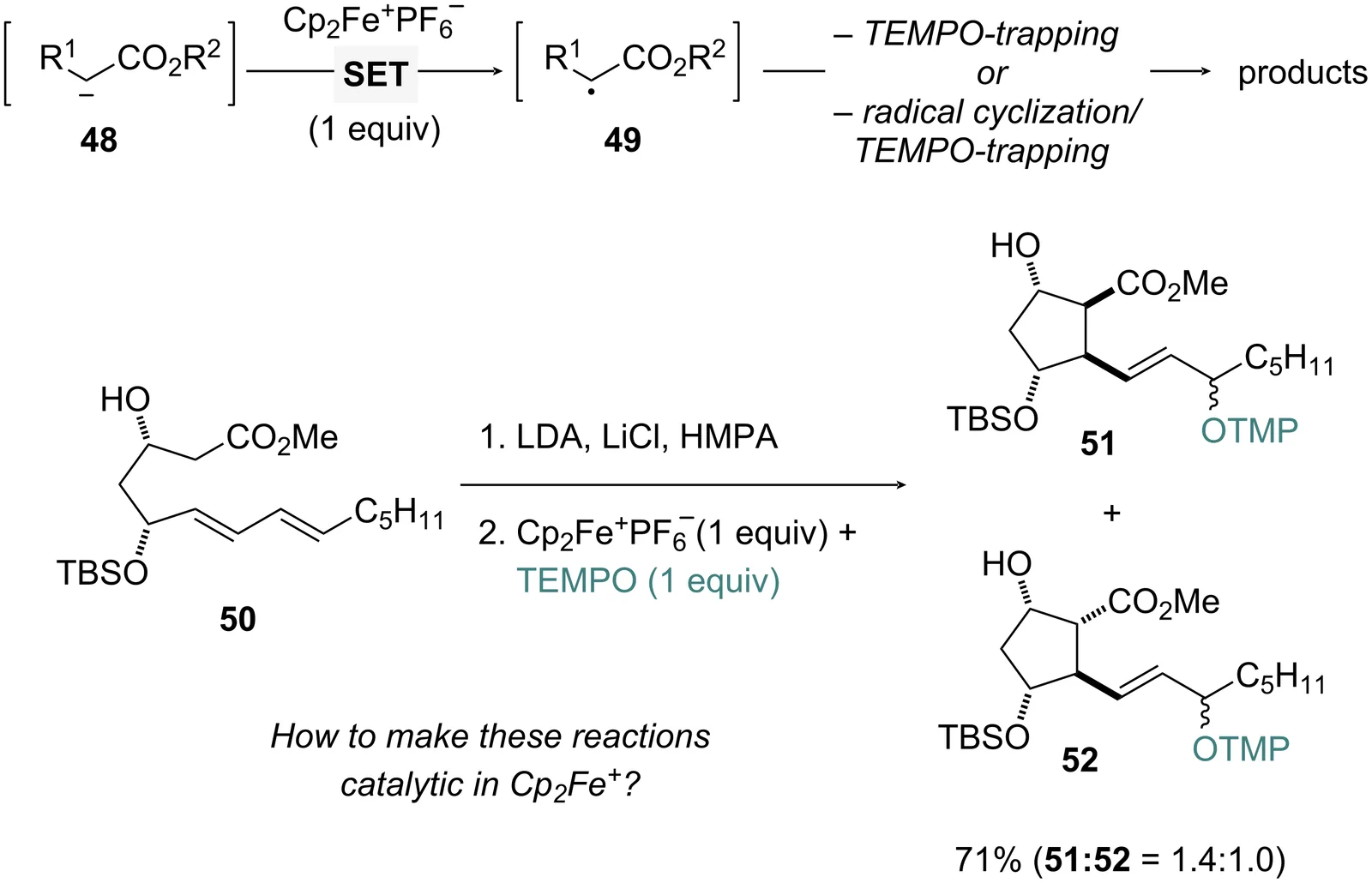
Heterointermediate reaction sequences developed by the Jahn group [67–68].

Ferrocenium salts, such as ferrocenium hexafluorophosphate (Cp_2_Fe^+^PF_6_^−^), were popularized as a SET oxidant by Jahn for a variety of anion-radical transformations, including the generation of α-carbonyl radicals **49** via SET oxidation of enolates **48** [65–66]. This approach was used for the oxidative C–C coupling of enolates, α-oxygenation of enolates, and tandem radical cyclization and radical trapping when conducted in the presence of TEMPO. The Jahn group applied this chemistry in a variety of settings, including the total synthesis of natural products (Scheme 10) [67–68]. One drawback of this approach was the stoichiometric use of the ferrocenium salt oxidant, which generates an equivalent amount of ferrocene as waste.

**Scheme 11:**
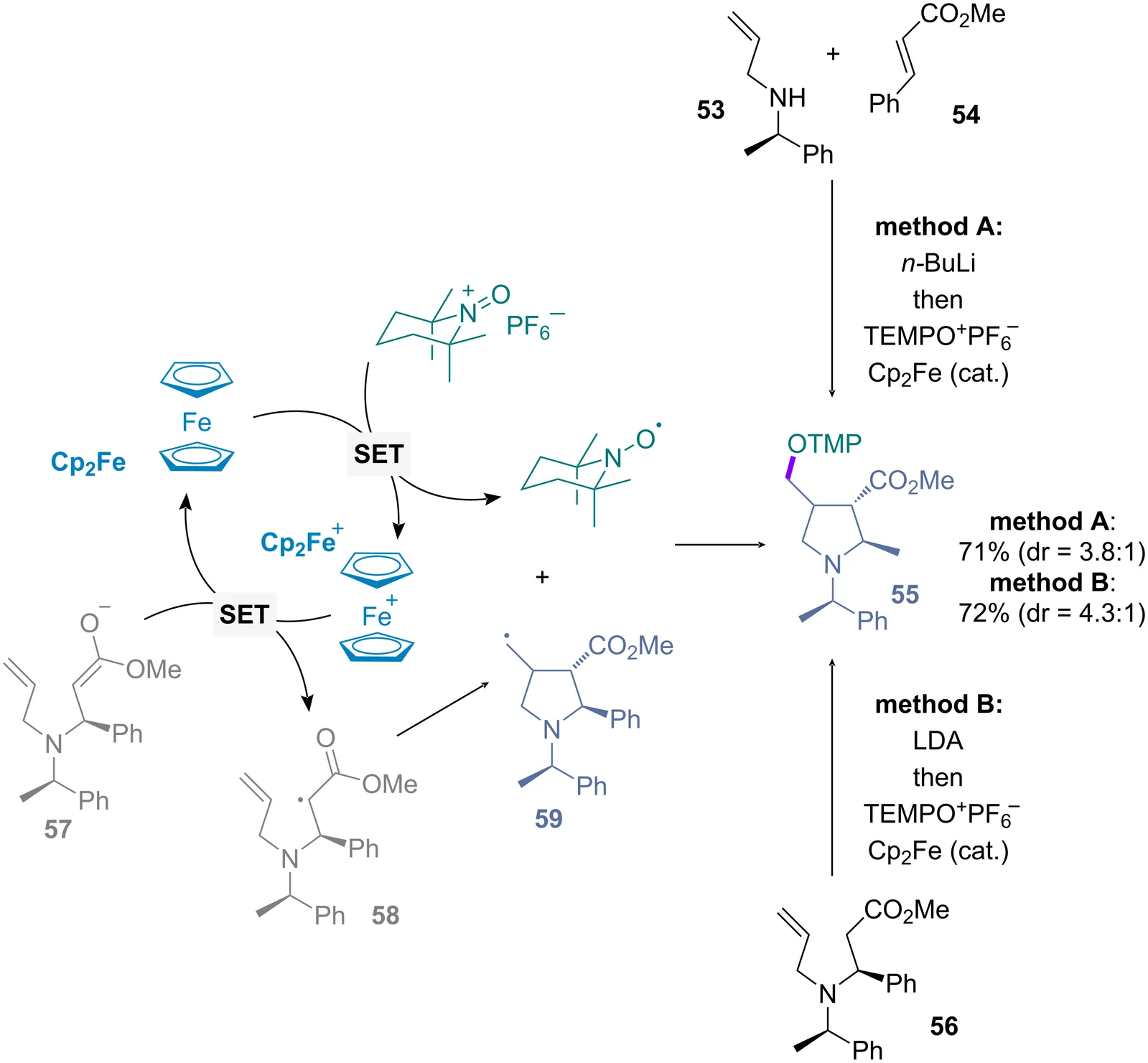
Fc/Fc^+^ catalysis driving the radical cyclization/oxygenation cascade and mechanistic rationale [69].

Recognizing this drawback and leveraging the greater reducing power of ferrocene than TEMPO, Jahn and co-workers have elegantly demonstrated that ferrocene can serve as a catalytic redox mediator when the reactions were conducted in the presence of a stoichiometric amount of TEMPO^+^ salt (Scheme 11) [69]. In this approach, mixing stoichiometric TEMPO^+^PF_6_^−^ with catalytic ferrocene (Cp_2_Fe) generates TEMPO and Cp_2_Fe^+^PF_6_^−^, the latter performing a SET-oxidation of enolate **57** to α-carbonyl radical **58**. During the SET-oxidation of **57** to **58** the catalytically used ferrocenium salt regenerates ferrocene thereby closing the catalytic cycle. The α-carbonyl radical **58** can either be trapped by TEMPO or be trapped after a rapid radical cyclization (**58** to **59** to **55**). As such, TEMPO is produced in a catalytic cycle and is immediately consumed, whereas ferrocene is used only in catalytic amounts. It is noteworthy that two approaches could be used to reach the enolate anion **57** – a direct deprotonation of a corresponding starting material **56** (method B) or in situ generation (method A). Method A is based on an earlier work by Davies, who showed that chiral 1-phenethylamine-based lithium amides can undergo a highly diastereoselective conjugate addition to cinnamate esters [70].

**Scheme 12:**
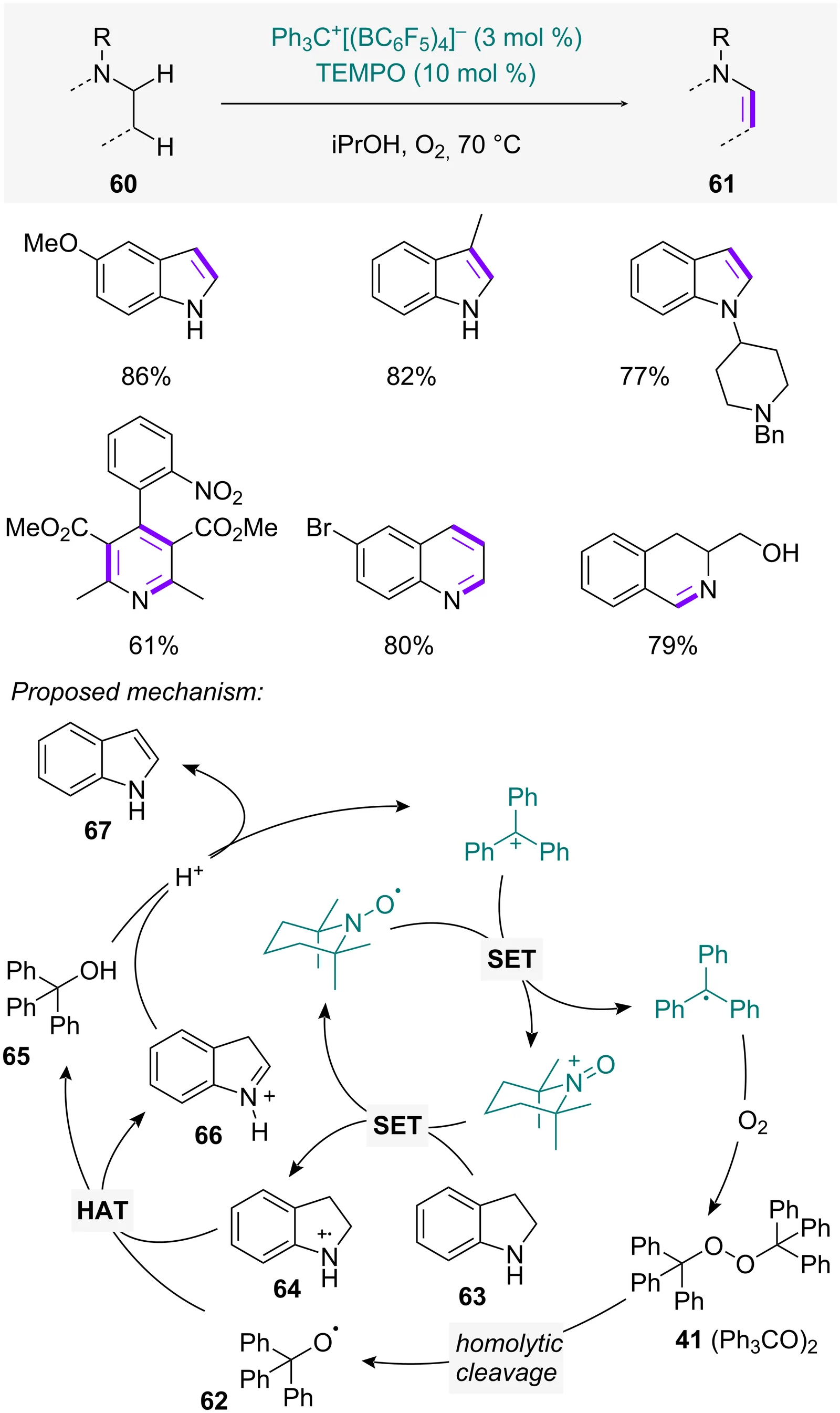
Cooperative Ph_3_C^+^/Ph_3_C• and TEMPO/TEMPO^+^ catalysis for aerobic dehydrogenation of N-heterocycles and the proposed mechanism [71].

As already mentioned above, the SET reduction of the trityl cation by TEMPO in the presence of molecular oxygen leads to the irreversible formation of trityl peroxide **41** (see Scheme 7). The trityl peroxide **41** is thermally labile and can undergo homolytic cleavage of the peroxy bond, generating two equivalents of the triphenylmethoxyl radical Ph_3_CO• **62**. Recently, Loh and co-workers for the first time leveraged this unique redox reaction between TEMPO and the trityl cation to achieve the cooperative dehydrogenation of saturated nitrogen heterocycles (Scheme 12) [71]. Remarkably, this aerobic dehydrogenation proceeds with low catalytic loadings of both the triphenylmethylcarbenium salt and TEMPO in the alcoholic solvent isopropanol, which neither deactivates the triphenylcarbenium cation nor participates in the dehydrogenation itself. According to the proposed mechanism, while TEMPO serves as a SET reductant to reduce triphenylcarbenium to the triphenylmethyl radical, it is recycled by a SET during the oxidation of the amine substrate **63** to the aminium radical cation **64**. The aminium radical cation **64** in turn engages in a hydrogen atom transfer (HAT) process with the triphenylmethoxyl radical Ph_3_CO• (**62**), resulting in the formation of iminium species **66** and triphenylmethanol (**65**). Deprotonative tautomerization of **66** to indole (**67**) and acidic ionization of triphenylmethanol to the triphenylcarbenium cation close the catalytic cycle. Interestingly, the reaction proceeds best in isopropanol, which would be expected to quench the catalytically generated species such as TEMPO^+^ (via oxidation to acetone) and triphenylmethoxyl radical **62** (via HAT).

### Redox catalysis by persistent radicals

**Figure 5:**
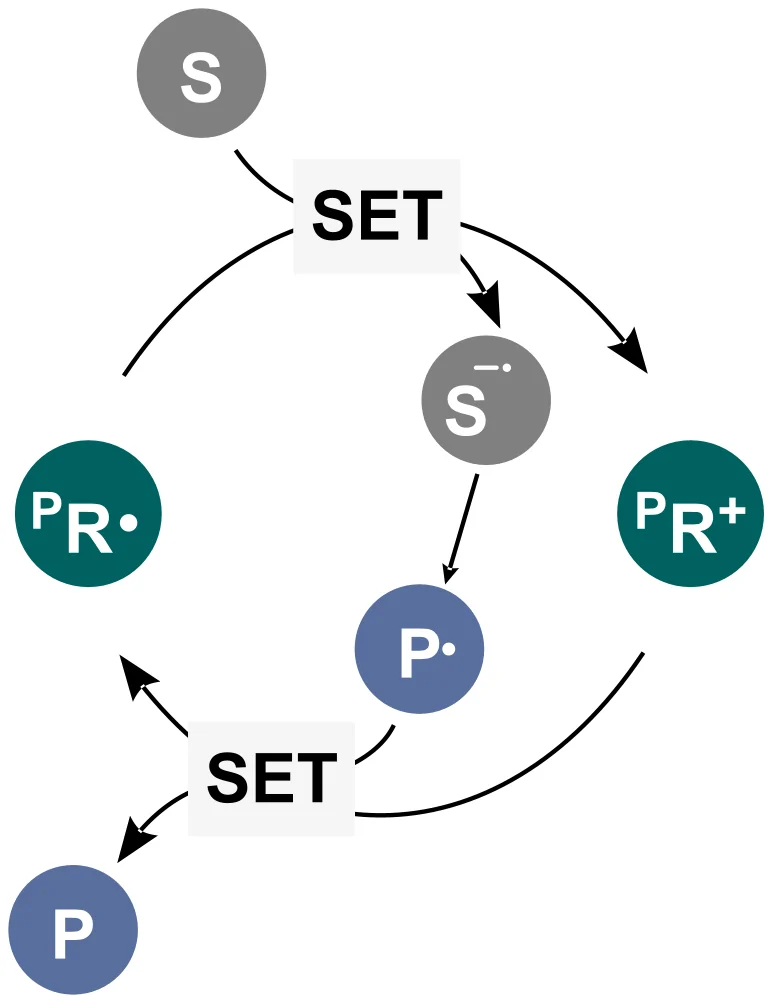
Conceptualization of persistent radicals as single-electron shuttle catalysts in redox-neutral transformations.

The redox-active properties of persistent radicals could enable them to serve as ground-state single-electron shuttle catalysts in metal-free processes (Figure 5). However, there are only limited classes of substrates that can be reduced by traditionally used persistent nitroxide radicals.

**Scheme 13:**
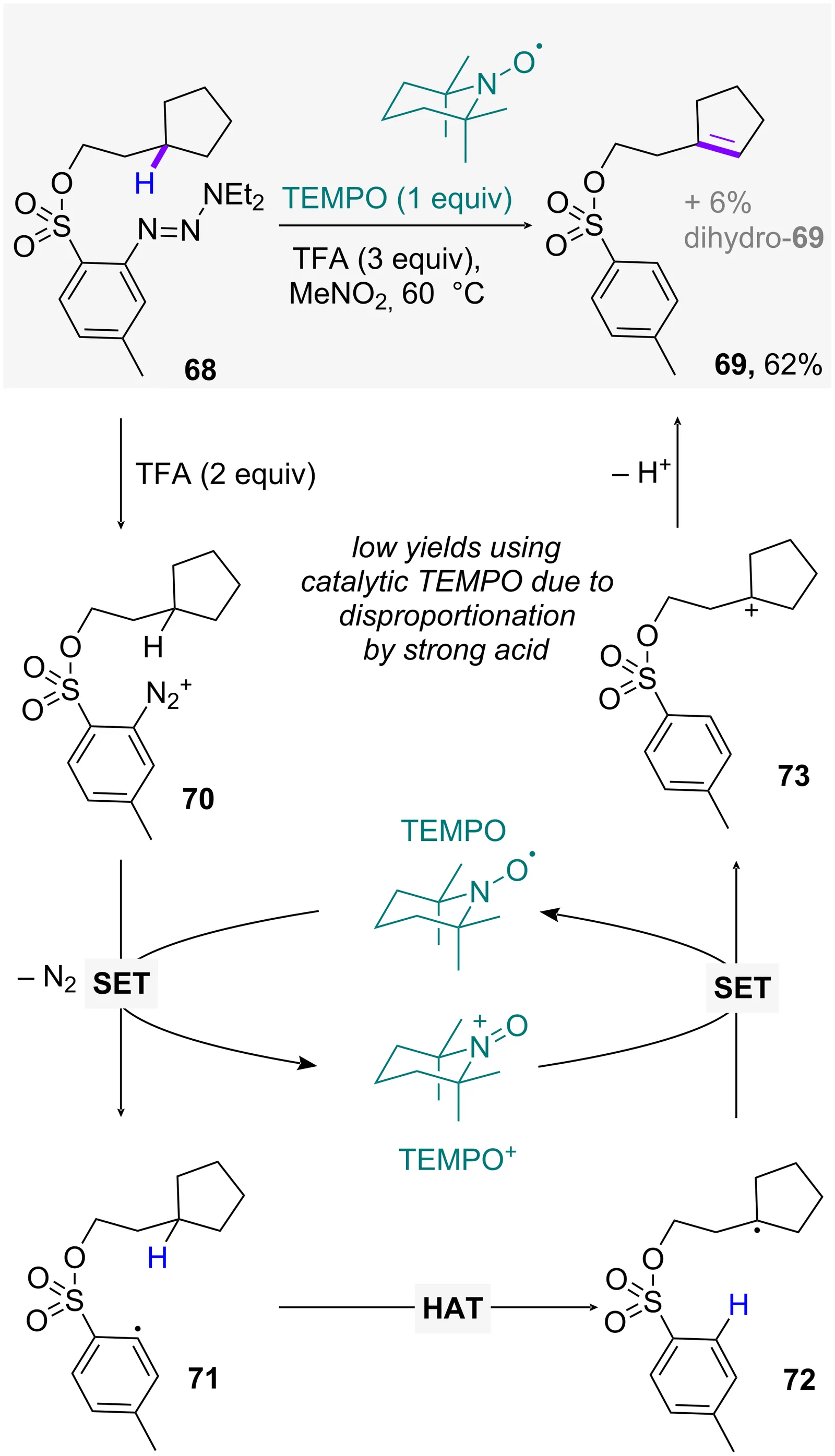
TEMPO/TEMPO^+^ single-electron shuttle in radical-polar crossover reaction [72].

The potential of TEMPO to act as a single-electron shuttle in catalytic redox reactions was first demonstrated by Baran and co-workers (Scheme 13), albeit with low turnover numbers (TON ≤ 3) [72]. They used TEMPO to mediate radical-polar crossover steps in alkane desaturation reactions guided by a *o*-triazyltosyl directing group. However, the practical yields were achieved only when TEMPO was used in stoichiometric amount. This was attributed to the instability of TEMPO under strongly acidic conditions, which promotes rapid disproportionation of nitroxides. The products obtained from this method were often inseparable mixtures of the alkene and the alkane ranging from 20:1 to 7:1. In addition to the discussed mechanism, the possibility of trapping radical **72** by TEMPO followed by TEMPOH elimination can also give the product. However, the authors of the article did not mention any detection of TEMPO adducts.

**Scheme 14:**
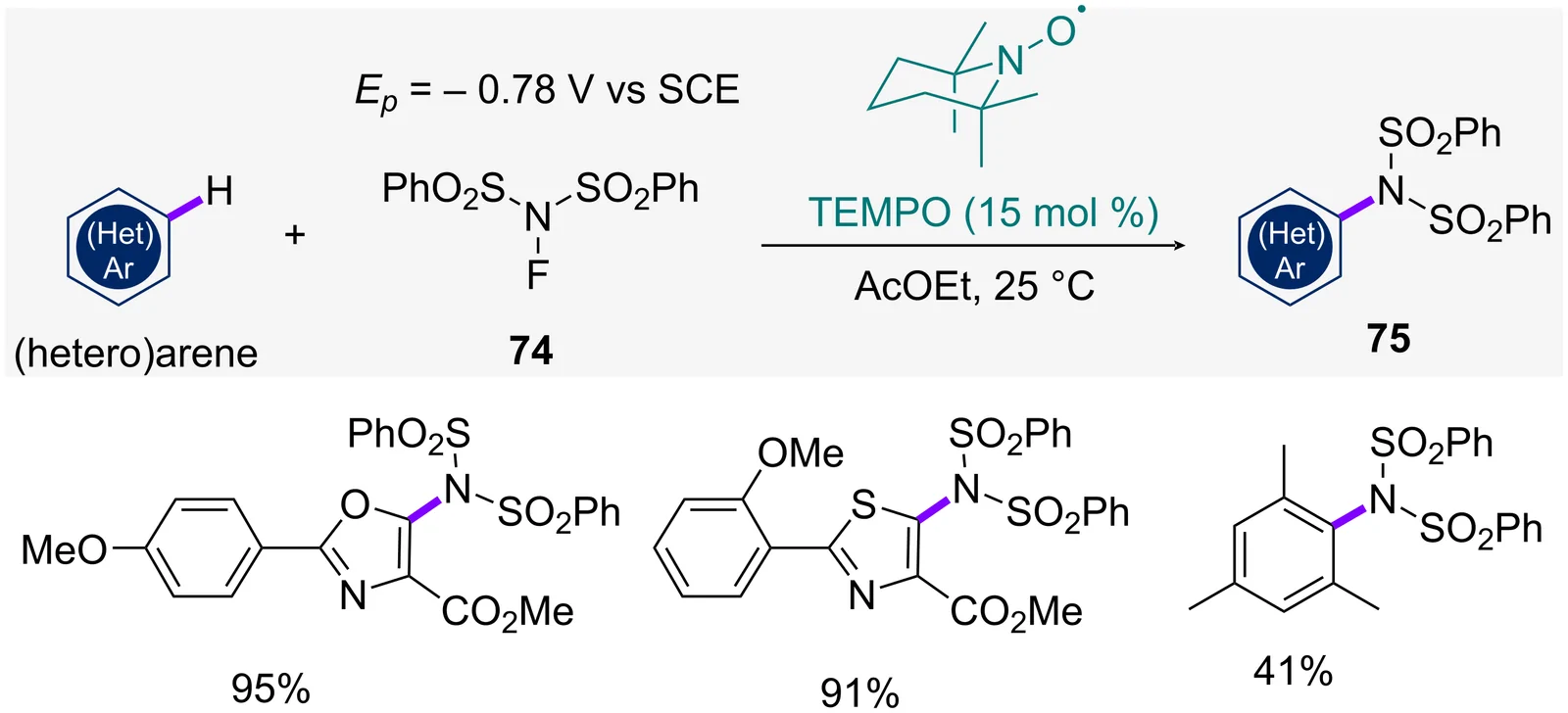
Redox-neutral C–H amination of (hetero)arenes catalyzed by TEMPO [73].

The Li group reported that *N*-fluorobenzenesulfonimide (NFSI), despite its relatively negative redox potential, can be reduced by TEMPO, thereby is applicable for aminating (hetero)arenes (Scheme 14) [73].

**Scheme 15:**
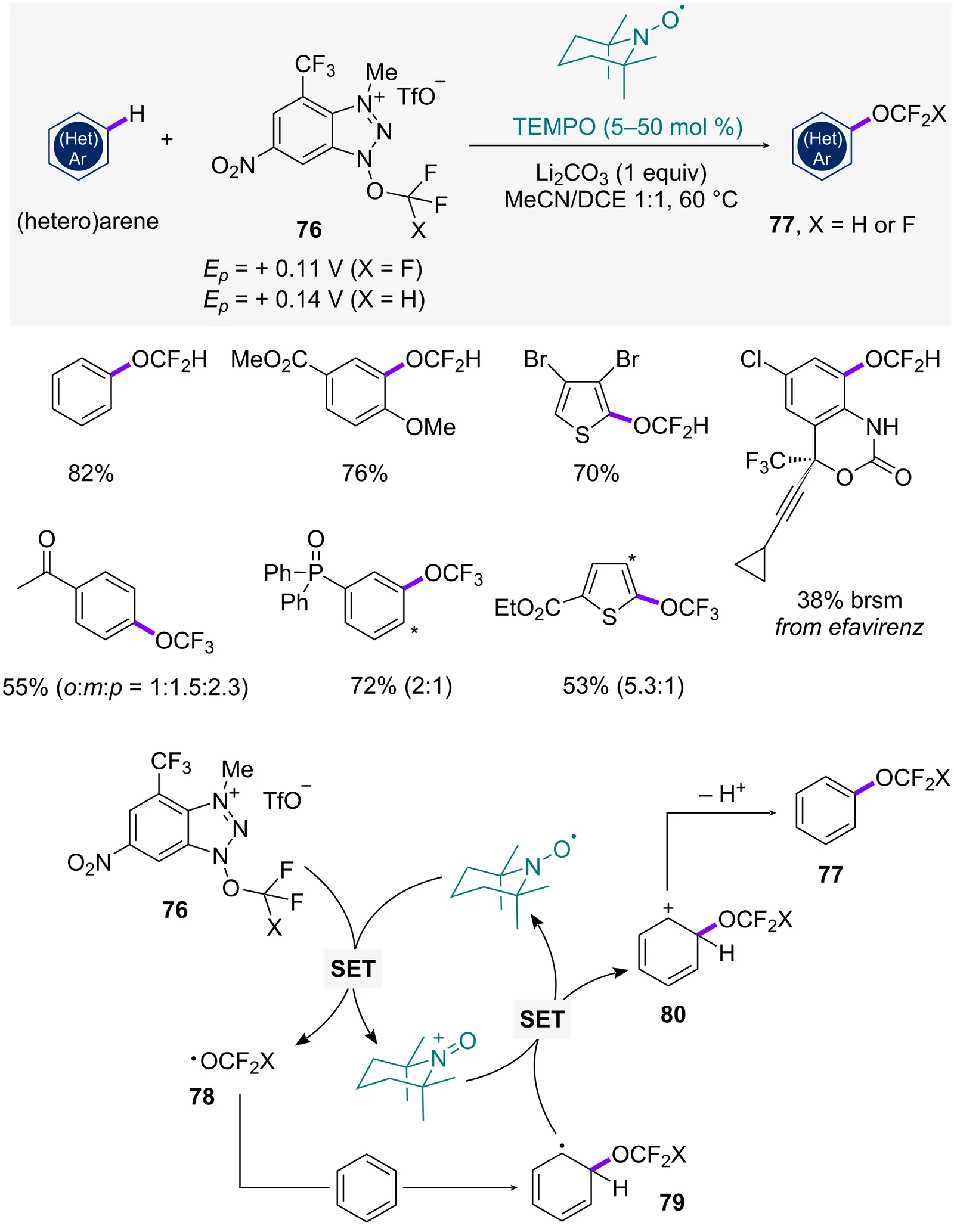
Redox-neutral C–H di- and trifluoromethoxylation of (hetero)arenes catalyzed by TEMPO [74].

The Ngai group also reported a TEMPO/TEMPO^+^ catalytic system as a single-electron shuttle for the intermolecular C–H di- and trifluoromethoxylation of (hetero)arenes (Scheme 15) [74]. Impressively, the efficiency of the TEMPO single-electron shuttle catalysis was comparable to a photoredox catalysis protocol using Ru(bpy)_3_(PF_6_)_2_, reported earlier by the same group using the same di- and trifluoromethoxylation reagents [75]. Although, the base-induced homolytic aromatic substitution (BHAS) chain-transfer pathway cannot be excluded, it appears unlikely given the superior performance under heterogeneous conditions. Under otherwise identical conditions, the homogeneous base 2,6-di-*tert*-butyl-4-methylpyridine afforded the product in only 39% yield.

**Figure 6:**
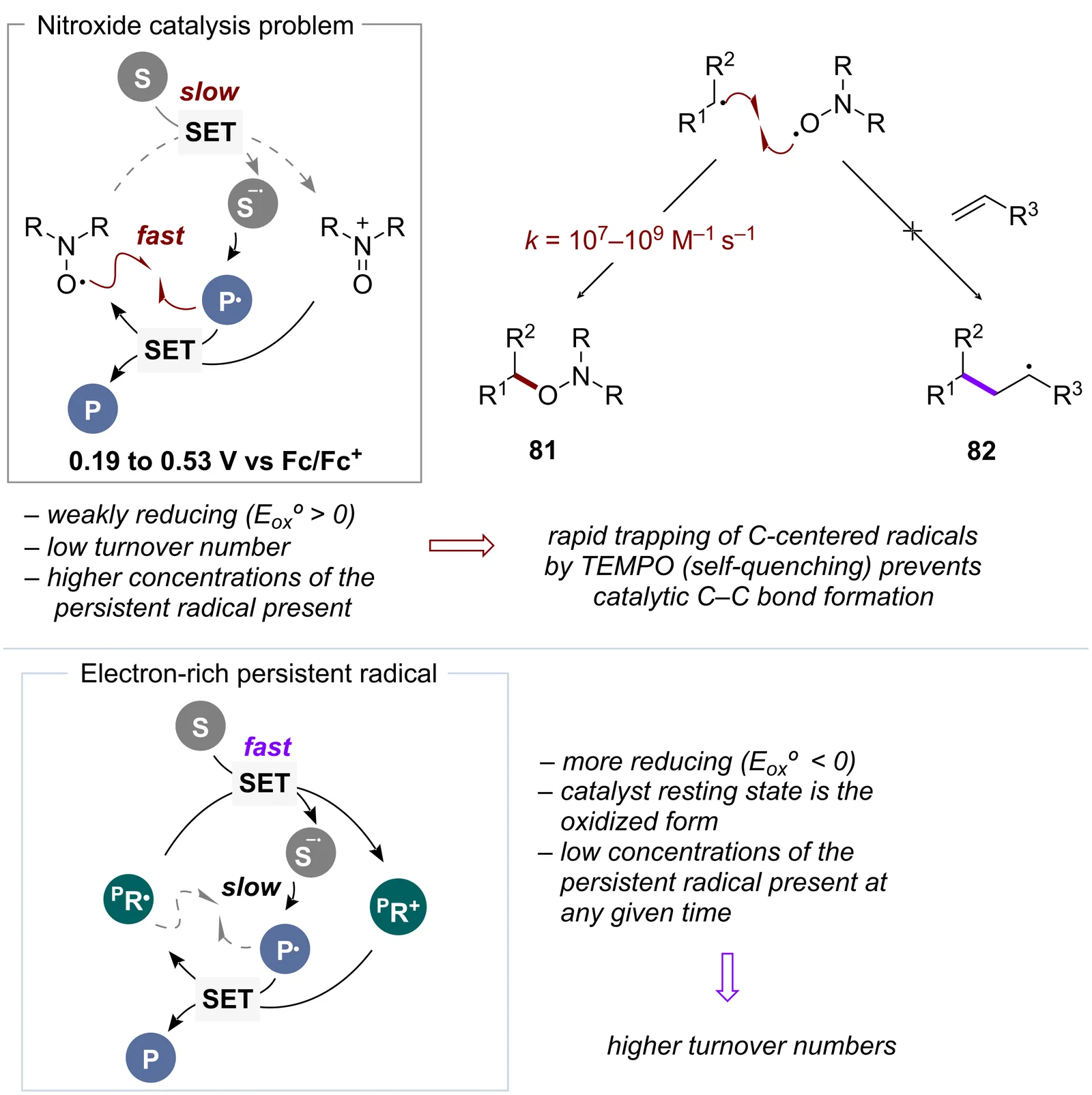
Challenges in catalytic C–C bond formation with nitroxide/oxoammonium catalysis and a strategy to overcome it with more reducing persistent radicals.

As evidenced by the examples covered thus far in this review, the applications of nitroxides and their derivatives as redox mediators/catalysts have largely dominated those of other classes of persistent radicals. One can expect that other classes of persistent radicals could also offer considerable promise in promoting and catalyzing transformations that are beyond the reach of nitroxides. One example of such a challenging catalytic transformation inaccessible to nitroxides is the *intermolecular* radical C–C bond formation. Because persistent radicals typically trap transient *C*-centered radicals with very fast kinetics [25–27], such reactions are prone to self-quenching and can be used as a radical clock in the determination of rate constants for radical addition to various alkenes [76]. Indeed, TEMPO, despite its ability to reduce arenediazonium salts, fails to catalyze the Gomberg–Bachmann–Hey reaction (C–H arylation of arenes with arenediazonium salts) due to rapid trapping of aryl radicals. Since nitroxides are only weakly reducing, the SET reduction of arenediazonium salts is slow, and both TEMPO^+^ and TEMPO coexist along with the transient *C*-centered radicals (Figure 6, top). The latter two cross-couple efficiently and selectively, halting further turnover. The success of Li’s TEMPO-catalyzed amination and Ngai’s TEMPO-catalyzed trifluoromethoxylation can thus be explained by the participation of heteroatom-centered radicals that do not couple with TEMPO due to electronic frustration.

Therefore, when using persistent radicals as single-electron shuttles in intermolecular C–C-bond-forming reactions, the generation of substrate radicals and the oxidative consumption of the persistent radical must proceed concurrently; i.e., the SET reduction of substrates by persistent radicals must be very fast. Using electron-rich persistent radicals could offer a potential solution to this problem (Figure 6, bottom).

Arenediazonium salts are among privileged precursors of aryl radicals [77–78]. Traditionally, aryl radical generation from arenediazonium salts has been carried out mainly using low-valent transition-metal salts such as Cu(I), Fe(II), and Ti(III) [79]. The developments in photoredox catalysis have enabled more sustainable approaches to harnessing the potential of arenediazonium salts in radical transformations that proceed via aryl radicals. More recently, mechanochemical and mechanoredox approaches have also been developed as complementary approaches [8–9]. The latter approach has the added advantage of mitigating the potential explosivity of arenediazonium salts [80–81].

**Scheme 16:**
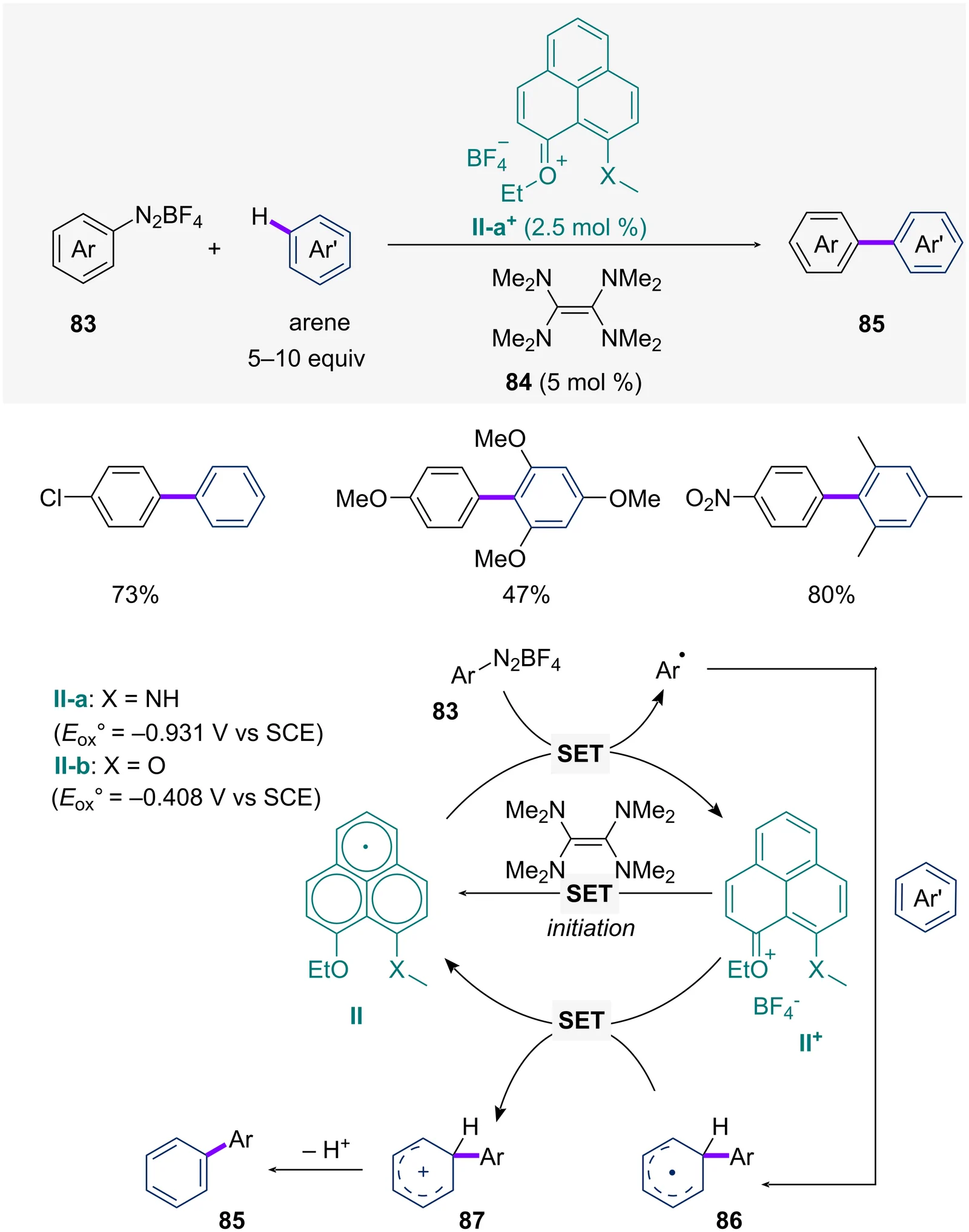
Phenalenyl radical-catalyzed C–H arylation of arenes [82].

However, examples of ground-state redox catalysis, even using easy-to-reduce arenediazonium salts and purely organic catalysts, are scarce. In this regard, Mandal and co-workers reported that phenalenyl radicals can serve as organic redox catalysts for the C–H arylation of arenes with arenediazonium salts (Scheme 16) [82]. The authors proposed a catalytic phenalenyl radical/cation (**II**/**II^+^**) cycle in which **II** reduces arenediazonium salts **83** by SET, while Lewis acid–like association of **II^+^** with cyclohexadienyl radical **86** facilitates the otherwise unfavorable catalyst-regenerating SET. The redox potential of phenalenyl radicals can be tuned. For example, phenalenyl radical **II-a** with a methylamino substituent is substantially stronger reducing than its methoxy analog **II-b** (*E*_ox_° = −0.931 V and −0.408 V, respectively vs SCE) [83]. Due to their highly reducing nature and propensity for dimerization, phenalenyl radicals are not bench-stable and must be generated and stored under inert conditions. Indeed, in these transformations, phenalenyl radicals were generated in situ via SET reduction of phenalenyl cations **II^+^** using the organic super-electron donor TDAE (tetrakis(dimethylamino)ethylene, **84**) [84].

As mentioned in the introduction, *N*-centered radicals such as Kuhn-type verdazyls (**V**) are bench-stable persistent radicals with stabilities comparable to nitroxides. Yet, they are significantly more electron-rich and stronger reductants, exceeding the reducing power of ferrocene, an established single-electron shuttle.

**Scheme 17:**
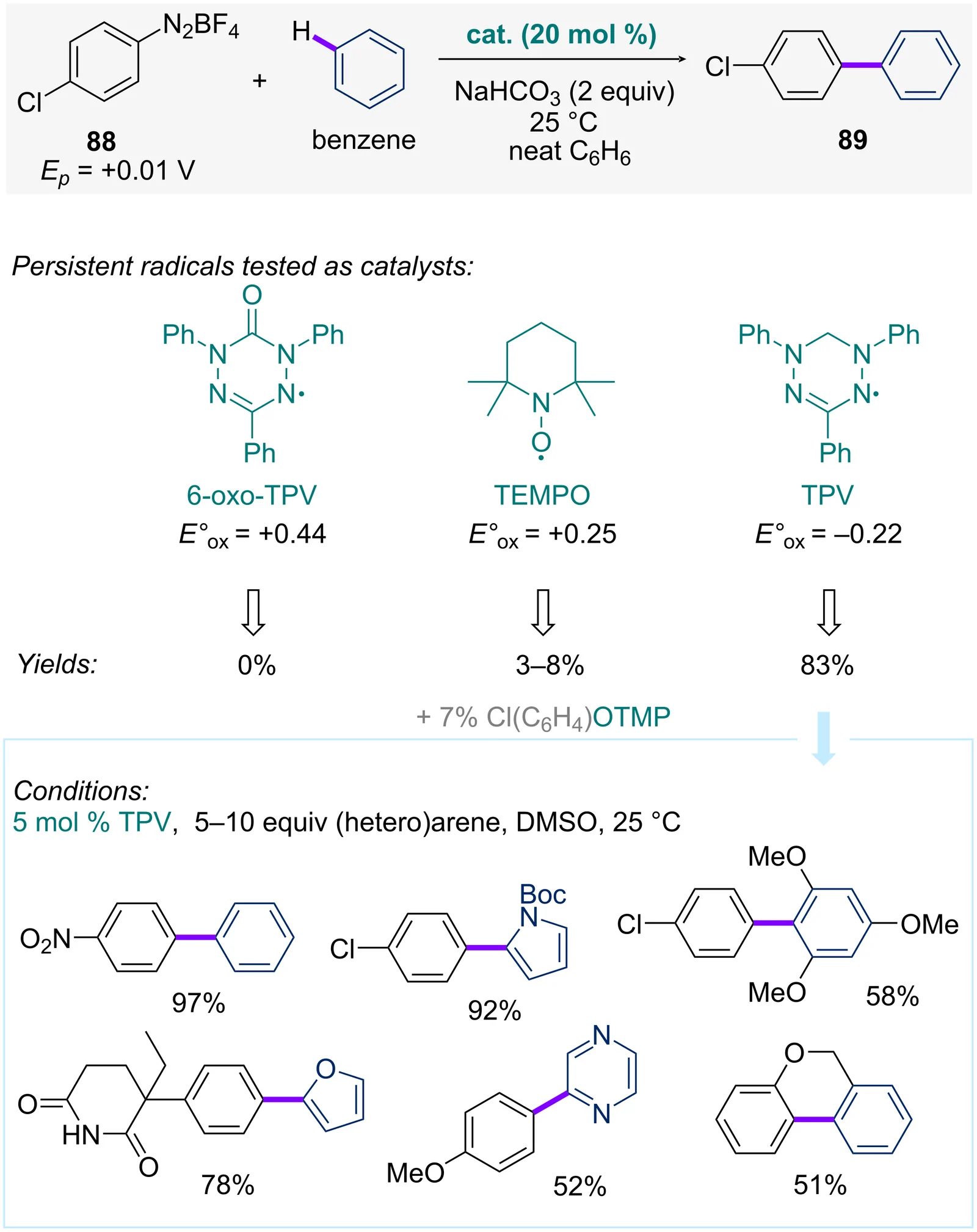
Redox-neutral C–H arylation catalyzed by triphenylverdazyl and its comparison to TEMPO and 6-oxo-TPV [85].

Amatov and co-workers recently developed an organocatalytic C–H arylation of arenes and heteroarenes (Scheme 17) [85] based on the overlooked precedent by Bogillo and Gragerov, who reported their ability to rapidly reduce arenediazonium salts. They developed an organocatalytic C–H arylation catalyzed by triphenylverdazyl (TPV). It was shown that Kuhn verdazyls are more efficient SET reductants and single-electron shuttle catalysts than nitroxides in these C–C bond-forming reactions. Comparative studies with TEMPO and electron-deficient 6-oxo-TPV further demonstrated TPV's unique catalytic efficiency in these transformations. In this regard, Kuhn verdazyls are more advantageous than phenalenyl radicals, which are not bench-stable and require in situ generation with super-electron donors or alkali metals.

**Scheme 18:**
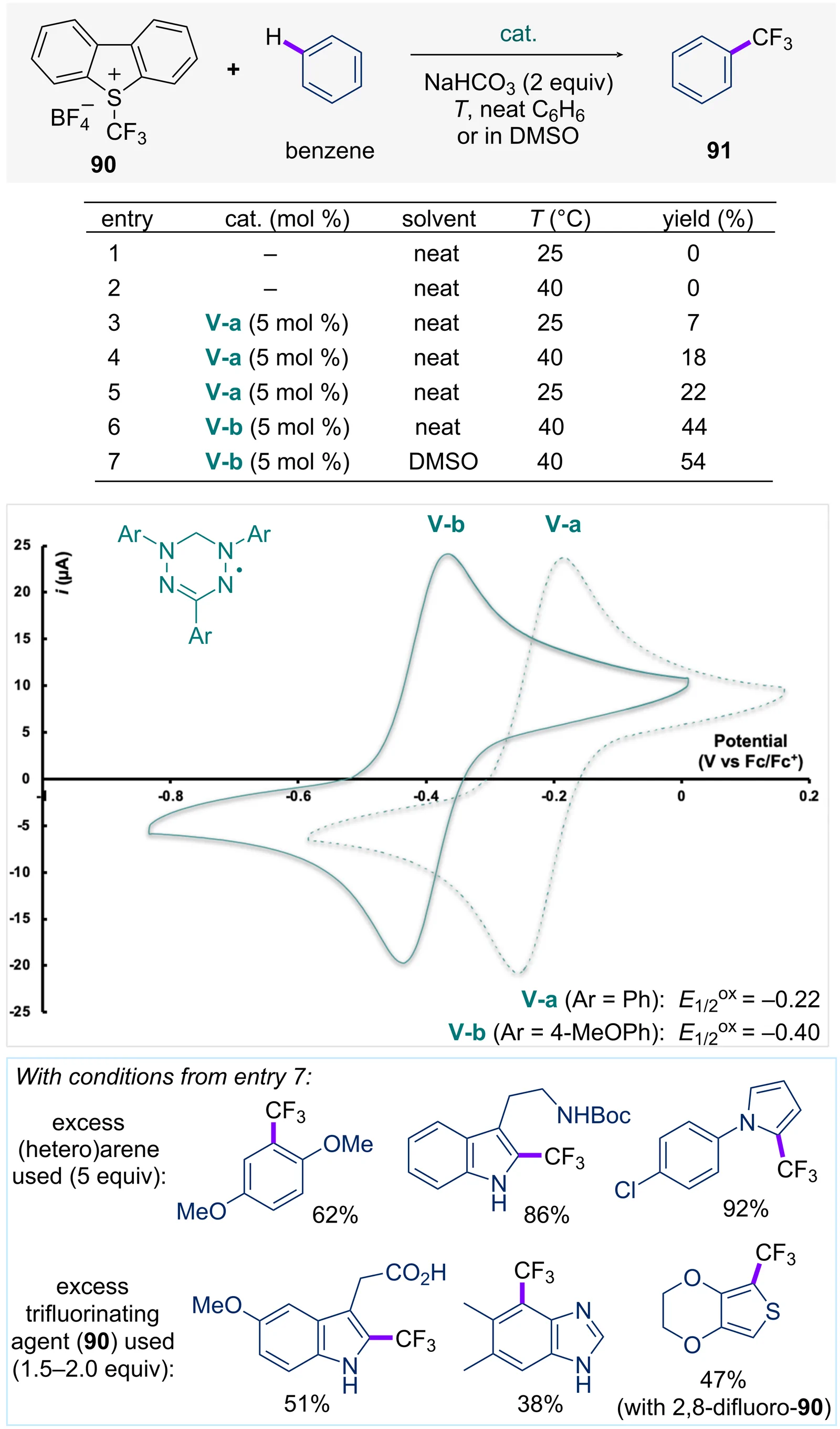
Redox tuning of Kuhn verdazyls for C–H trifluoromethylation. Cyclic voltammogram was reproduced from [85] (© 2025 S. Mujahed et al., published in Angew. Chem. Int. Ed. published by Wiley-VCH GmbH, distributed under the terms of the Creative Commons Attribution-NonCommercial-NoDerivs 4.0 License, https://creativecommons.org/licenses/by-nc-nd/4.0/). This content is not subject to CC BY 4.0.

Redox properties of Kuhn verdazyls and their oxo-analogs can be readily tuned by introducing electron-donating or electron-withdrawing groups into the aromatic groups [12]. Amatov and co-workers also demonstrated that the redox tunability of verdazyls allows activation of the Umemoto reagent **90**, which has a significantly negative redox potential compared to arenediazonium salts [85]. 4-Methoxyphenyl-substituted verdazyl **V-b**, with doubled reducing power, as shown by cyclic voltammetry, enabled a redox-neutral C–H trifluoromethylation of arenes and heteroarenes (Scheme 18).

**Scheme 19:**
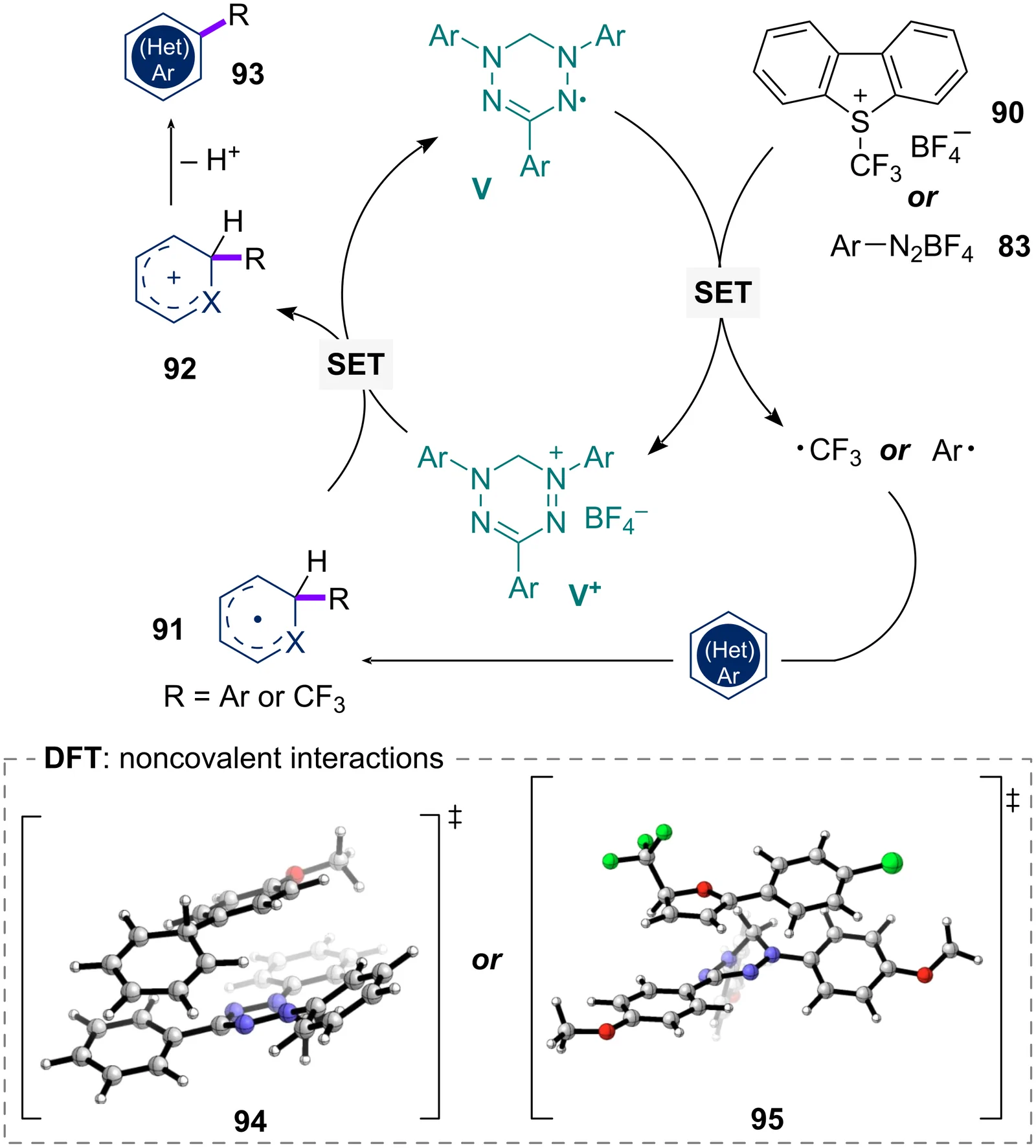
Generalized mechanism of verdazyl-catalyzed C–H arylation and C–H trifluoromethylation. DFT-derived structures was reproduced from [85] (© 2025 S. Mujahed et al., published in Angew. Chem. Int. Ed. published by Wiley-VCH GmbH, distributed under the terms of the Creative Commons Attribution-NonCommercial-NoDerivs 4.0 License, https://creativecommons.org/licenses/by-nc-nd/4.0/). This content is not subject to CC BY 4.0

Based on experimental and computational studies, the mechanism of verdazyl-catalyzed C–H arylation and C–H trifluoromethylation was proposed to commence with the fast SET reduction of onium salts (aryldiazonium or Umemoto’s sulfonium salt) by verdazyls **V** to generate aryl or trifluoromethyl radicals, respectively (Scheme 19). This process is accompanied by the oxidation of verdazyl **V** to a verdazylium salt **V^+^**. The addition of aryl or trifluoromethyl radicals generates stabilized pro-aromatic cyclohexadienyl-type radicals **91** that in turn are oxidized by **V^+^** to close the catalytic cycle, generating carbocations **92**, which undergo deprotonation to provide aromatic products **93**. DFT calculations suggested stabilizing noncovalent interactions between **V^+^** and the cyclohexadienyl-type radicals preceding SET, thereby regenerating the catalyst as shown in **94** and **95**.

**Scheme 20:**
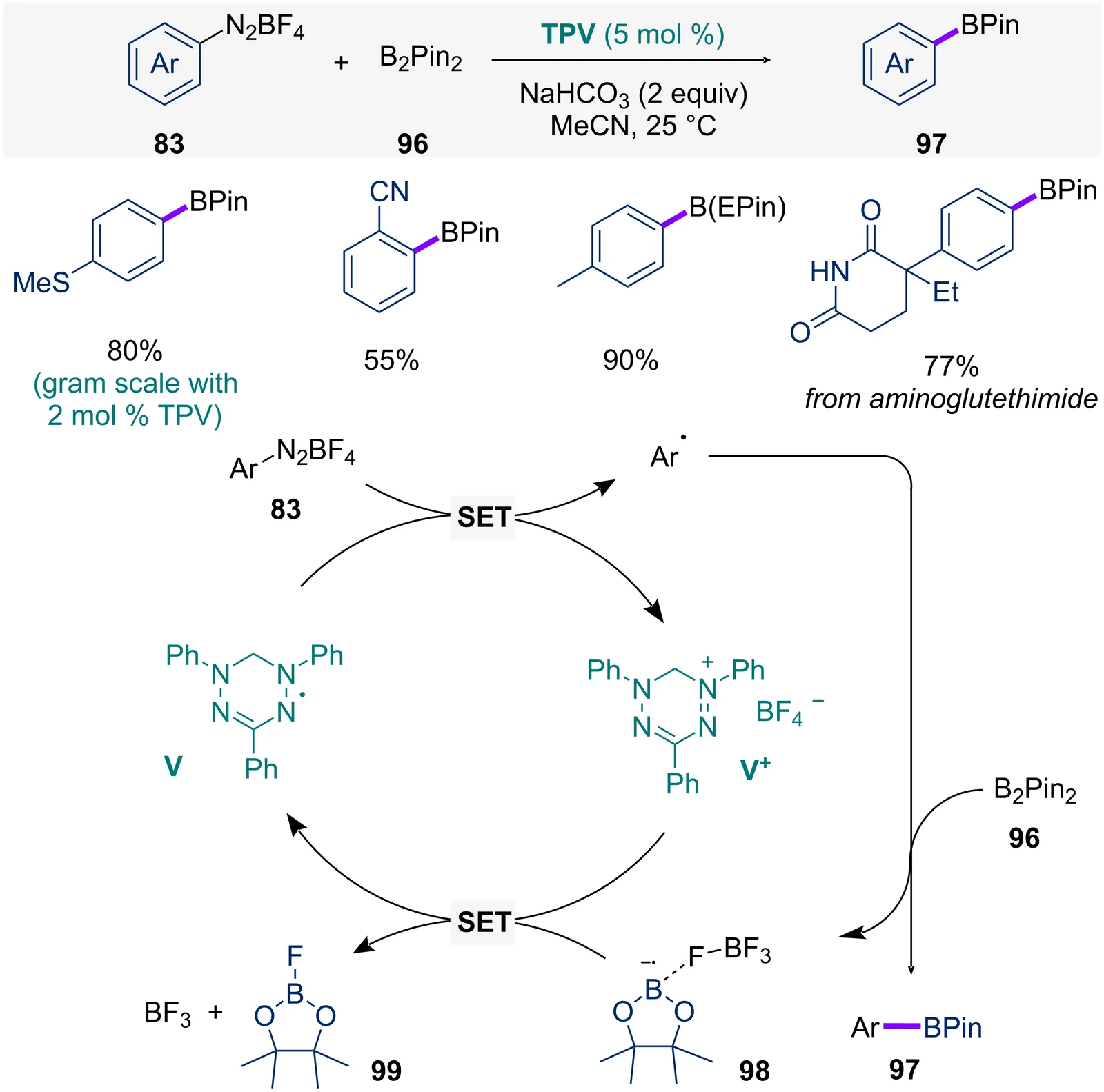
Verdazyl-catalyzed borylation of arenediazonium salts [86].

The unique organocatalytic potential of Kuhn verdazyls was also demonstrated in the Sandmeyer–Wang borylation of arenediazonium salts with B_2_Pin_2_ (Scheme 20) [86]. The performance of TPV was compared with other organic SET reductants. Interestingly, even in this reaction, which is generally accepted to proceed predominantly via a chain-propagation mechanism, TPV outperformed other promoters at room temperature, without light activation. Experimental and computational mechanistic studies rationalized the unique efficiency of TPV by showing that it acts as both a redox catalyst and a chain-repair agent, thereby overcoming the chain-propagation limitations typical of such transformations. The authors proposed and provided evidence supporting the preferential reduction of verdazylium salt **V^+^** over arenediazonium salts by the ligated boryl anion radical **98**.

**Scheme 21:**
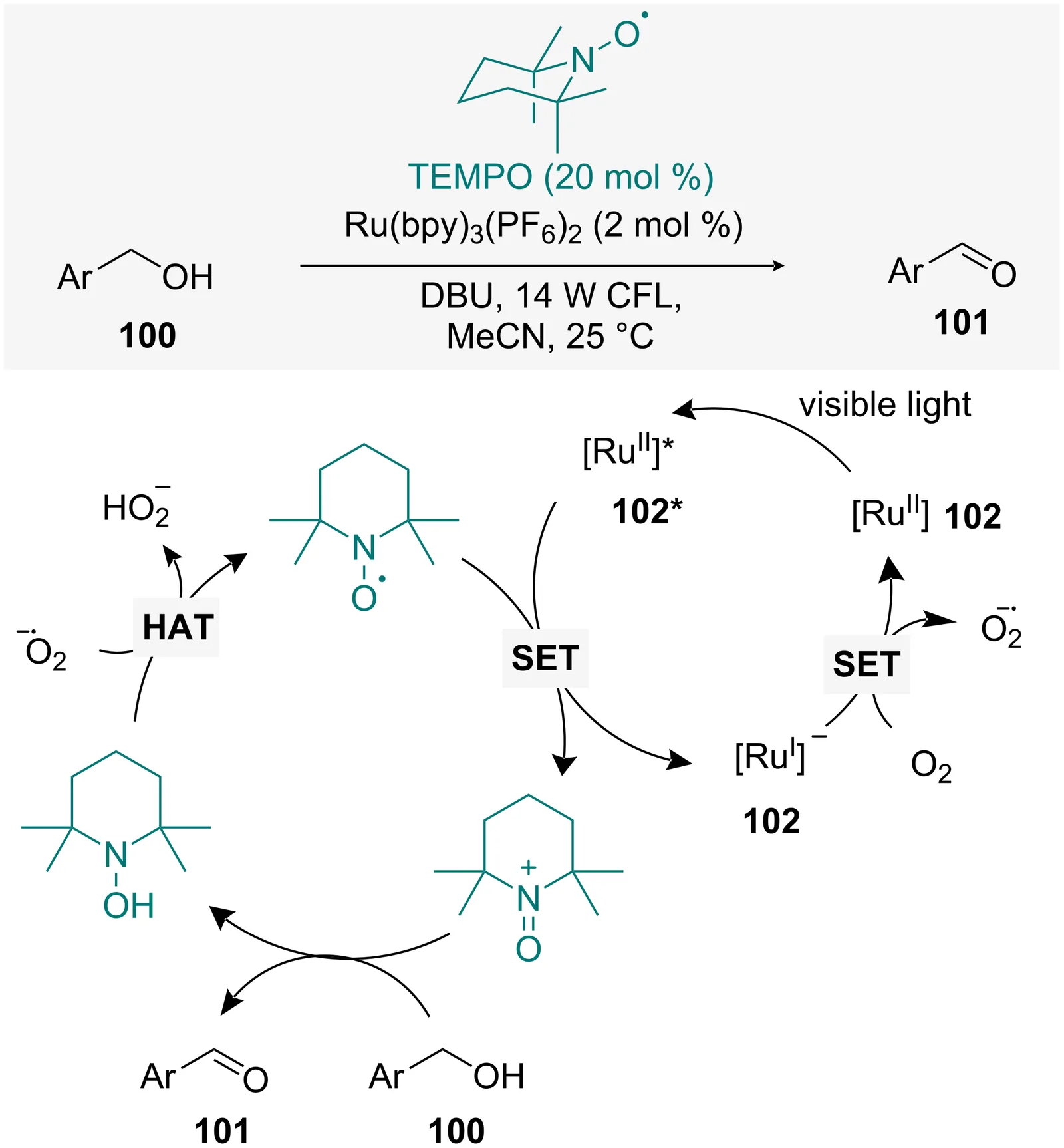
Visible-light-induced oxidation of alcohols by merging TEMPO and metal-complex photocatalysis [89].

The use of TEMPO and other *N*-oxyl species in various alcohol and amine oxidations, including in combination with transition metal catalysts and electrochemical processes, typically proceeds via two-electron processes, with the in situ-generated oxoammonium species acting as the oxidant that is continuously regenerated by reoxidation of TEMPOH, bypassing the regeneration of TEMPO, i.e., TEMPOH/TEMPO^+^ are involved in the catalytic cycle [87]. In another widely used method, such as the Stahl Cu/TEMPO-catalyzed aerobic alcohol oxidation protocol [88], a detailed mechanistic investigation revealed that a concerted hydrogen transfer from a Cu(II)–alkoxide to a coordinated nitroxyl species is more likely. As such, a TEMPO/TEMPOH redox couple is operative rather than TEMPOH/TEMPO^+^ and TEMPO/TEMPO^+^. In addition to these examples, there are other TEMPO-based oxidation methods in which it is proposed that all oxidation states (i.e., TEMPO, TEMPOH, and TEMPO^+^) are involved. For example, Li and co-workers reported an aerobic photoredox alcohol oxidation protocol using Ru(bpy)_3_(PF_6_)_2_ as photocatalyst and TEMPO as a cocatalyst (Scheme 21) [89]. According to the proposed mechanism, the active oxoammonium oxidant TEMPO^+^ is generated by SET oxidation with the excited Ru(II)* species (102*). TEMPO is recycled via a HAT reaction of TEMPOH with the superoxide anion radical that is in turn formed in the oxidation of Ru(I) to Ru(II). Diverse mechanistic scenarios of alcohol oxidations with TEMPO further underscore its utility and mechanistic versatility in redox transformations.

**Scheme 22:**
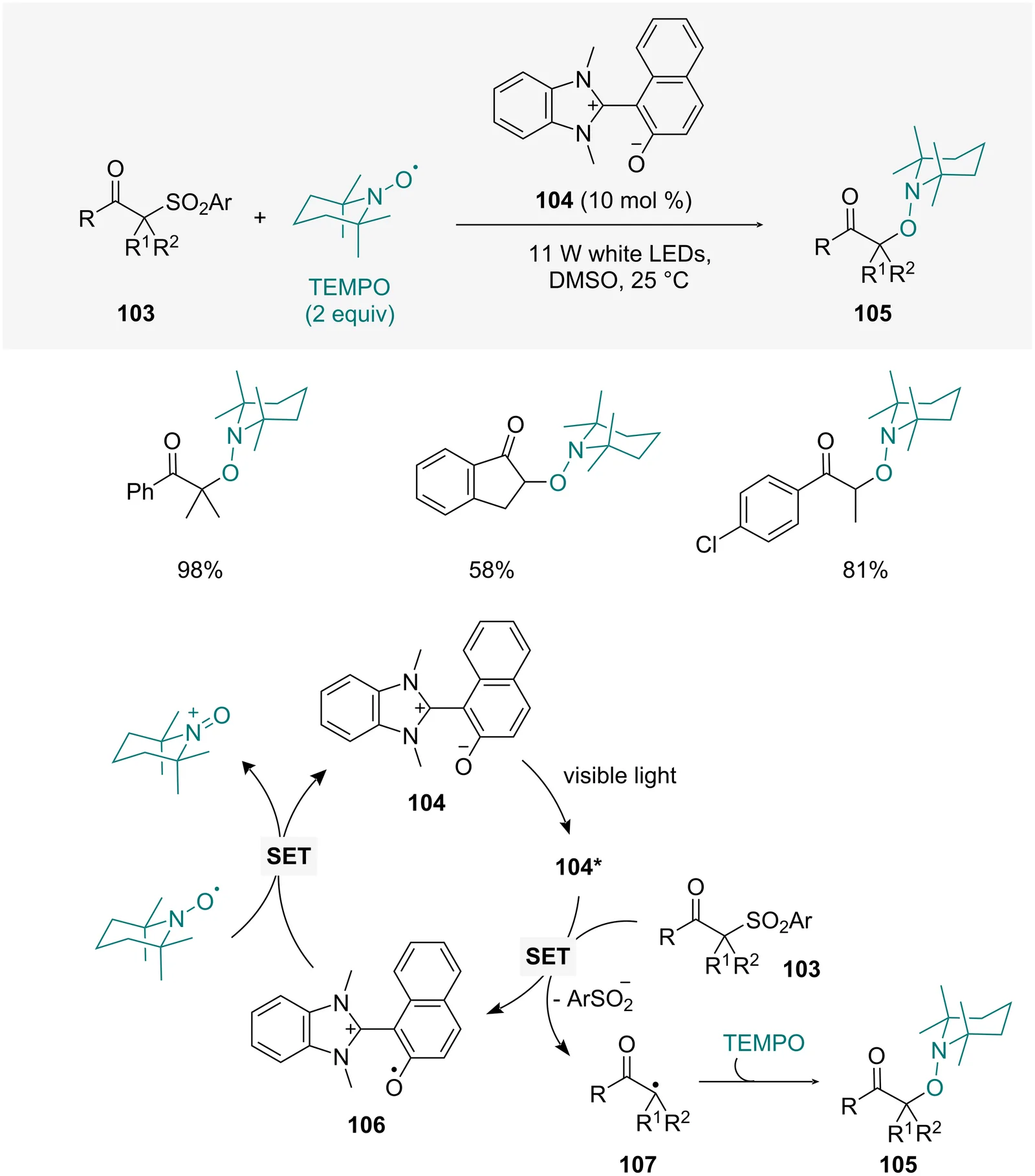
Benzimidazolium naphthoxide-catalyzed photo-desulfonylative α-oxyamination of α-sulfonylketones with TEMPO [90].

Hasegawa and co-workers reported a metal-free photocatalytic desulfonylative α-oxyamination of α-sulfonylketones **103** with benzimidazolium naphthoxide **104** as a photocatalyst (Scheme 22) [90]. They demonstrated that the photoexcited benzimidazolium naphthoxide 104* is a strong reductant capable of donating an electron to the α-sulfonylketone substrates. The radical anion formed in this manner loses a sulfinate anion to produce an α-ketoalkyl radical **107** that is trapped by TEMPO to generate the oxyamination product **105**. The photocatalyst is recycled in a SET process between TEMPO and the oxidized state of catalyst, **106**. In this transformation, TEMPO plays a dual role as both the terminal reductant in the catalytic cycle and a radical trapping reagent.

### Miscellaneous

**Scheme 23:**
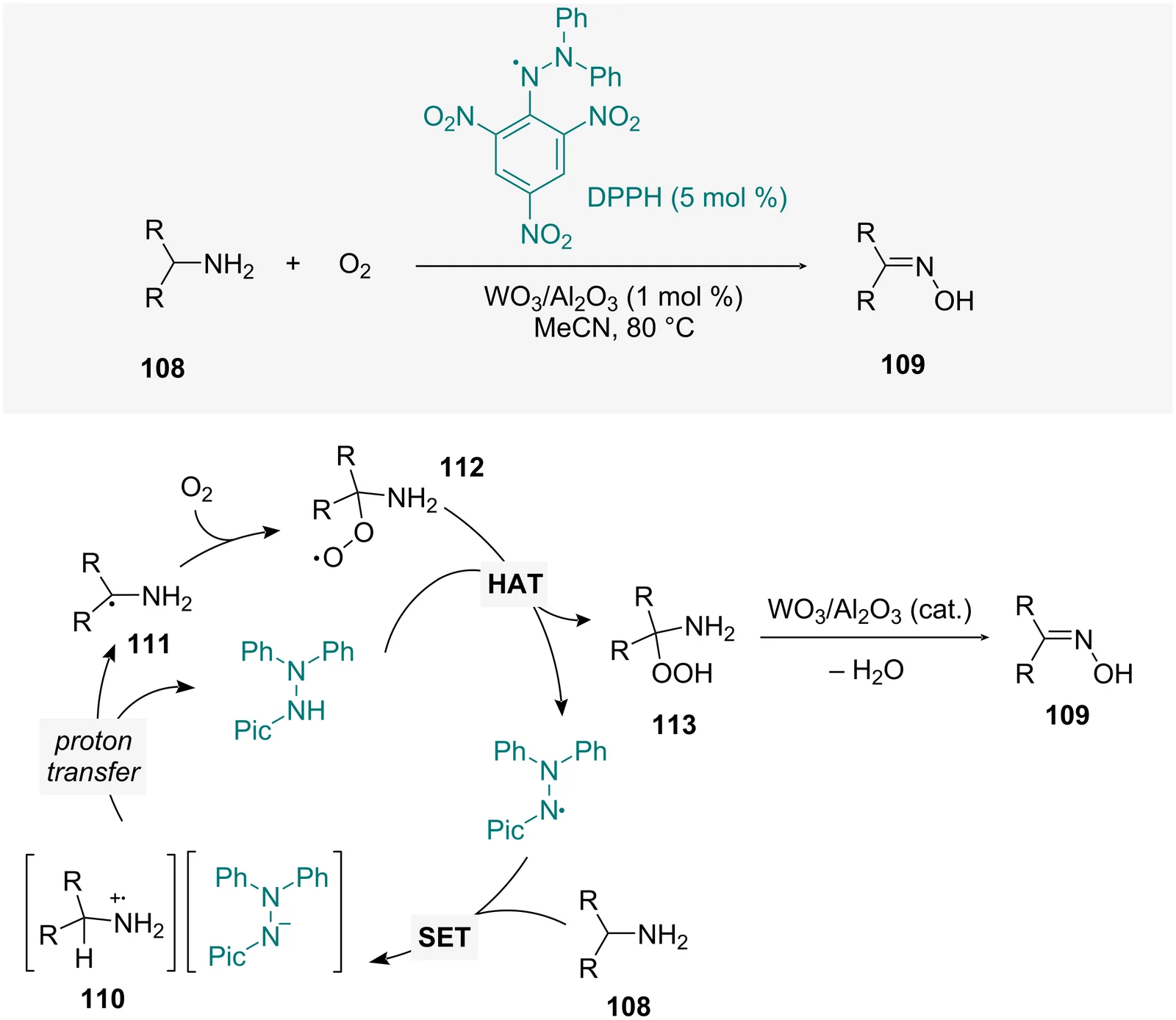
DPPH-catalyzed aerobic oxidation of primary amines [94].

Widely used as an analytical reagent, but relatively underutilized in synthetic chemistry, the *N*-centered persistent radical 2,2-diphenyl-1-picrylhydrazyl (DPPH) has long been used as an analytical tool in biological research. For a long time, its characteristic color change from purple to yellow upon reduction of the radical to the leuco form, DPPH-H, has been used in quantitative assays to evaluate the antioxidant activity of phenolic and other H-atom-donating compounds, including natural products. Despite decades of widespread use, the DPPH assay has not been fully standardized. Moreover, concerns have been raised regarding its reliability as a measure of antioxidant activity because the DPPH radical can be quenched through multiple pathways, including hydrogen atom transfer (HAT), proton-coupled electron transfer (PCET) and single electron transfer (SET) mechanisms, with the dominant process depending on factors such as solvent, pH, temperature, X–H bond strength, relative reactant concentrations, and the presence of oxygen [91]. The kinetic measurements suggest that DPPH quenching in alcoholic solutions often proceeds predominantly through SET from phenolate anions under these conditions, rather than via HAT or PCET [91–93]. An intriguing report by Murahashi’s group in 2008 shows that the DPPH radical can serve as an excellent catalytic SET oxidant for primary amines [94–95]. They demonstrated that primary amines can be catalytically oxidized to oximes with DPPH and a WO_3_/Al_2_O_3_ co-catalytic system with oxygen as the terminal oxidant (Scheme 23). According to the proposed mechanism, the SET oxidation of amines **108** by DPPH generates an ion-pair consisting of primary aminium radical **110** and DPPH^¯^, which collapses into the leuco form DPPH-H and an α-aminoalkyl radical **111**. The latter is trapped by molecular oxygen to form an α-aminoalkylperoxyl radical **112**, which then abstracts a hydrogen atom from DPPH-H, thereby regenerating the DPPH radical and simultaneously forming the α-aminoalkylhydroperoxide product **113**. Finally, peroxide **113** serves as a substrate for further dehydrative decomposition by the WO_3_ catalyst. A few years later, as a follow-up for this catalytic system’s other applications, Lu’s group reported the oxidation of alcohols [96]. Both benzylic and aliphatic primary alcohols under these conditions underwent selective oxidation to aldehydes (>90% selectivity, with the remainder being overoxidation to carboxylic acids). Bulky secondary alcohols provided ketones without any limitations caused by the steric environment.

As discussed in the preceding section, nitroxide radicals have steadily dominated the field of redox transformations as SET mediators or catalysts; hence, it is useful to look beyond TEMPO for additional examples. As one might expect, a myriad of such transformations has been reported, with other persistent radicals operating in the SET manifold, often without explicitly identifying the intermediates as persistent radicals. For this section, to take an expanded look at the scope of such reactions, the criteria for stability during storage as an isolated compound are relaxed. For example, even though the trityl radical was discovered by Gomberg in 1900 and is the first persistent radical known, the parent triphenylmethyl radical exists in equilibrium with its dimer and cannot be isolated as a purely monomeric species under ordinary conditions. Although many specially designed derivatives have been found to be kinetically stable enough to be isolated, stored, and handled as pure persistent radicals for months under air- and light-free conditions [19], their application development is underexplored. However, the entry into the SET manifold can be readily achieved through its other redox forms, as tritylium cations are known to be stable and can be stored.

**Scheme 24:**
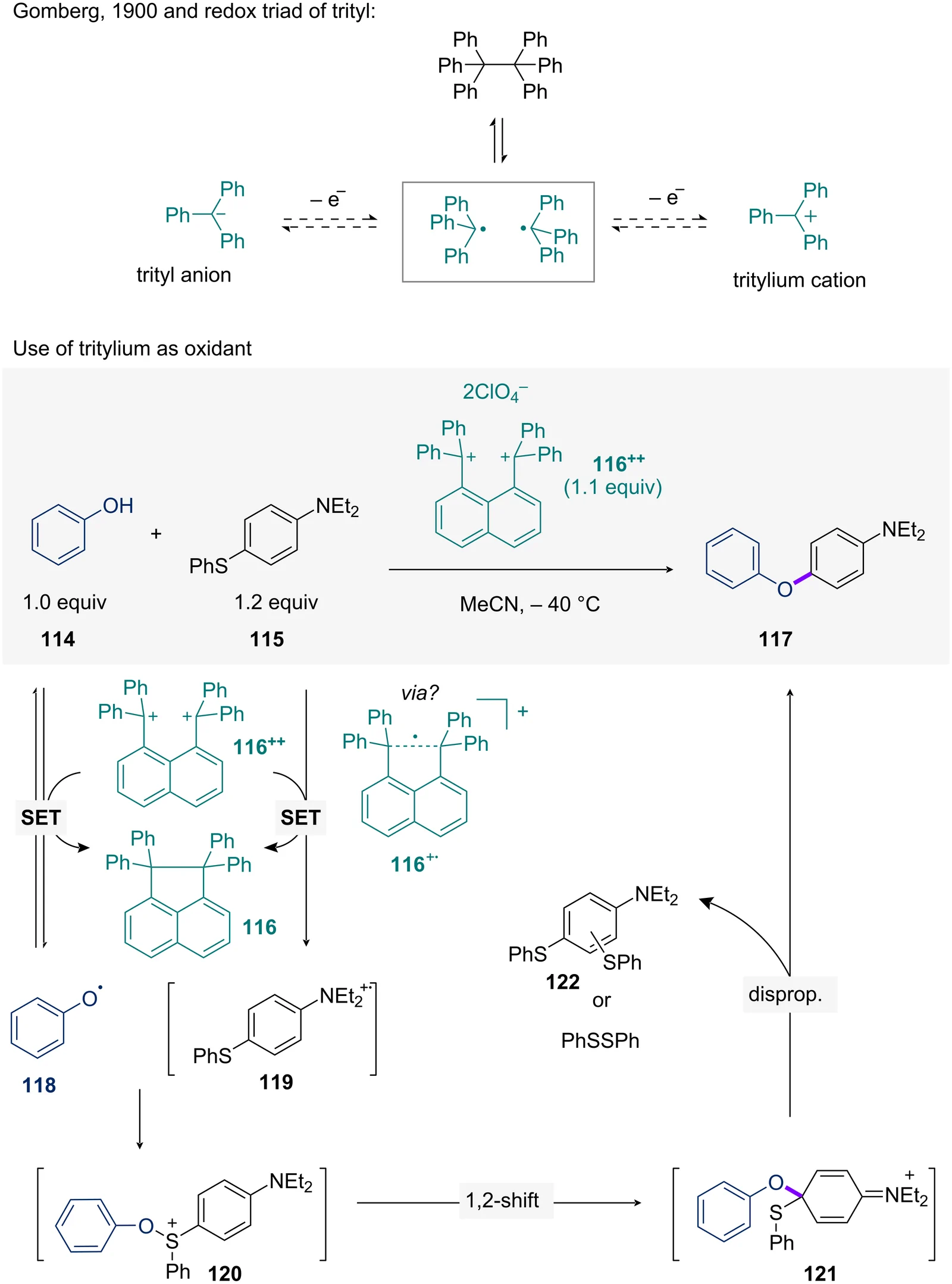
Bis(triarylmethylium)-mediated oxidative arylation of phenols [97].

In 2005, the Ichikawa group developed a unique dethiolative phenolation reaction with phenols and 4-thioarylanilines using a bis-tritylium salt **116^++^** (Scheme 24) [97]. The initial study of such bis-trityliums was reported by Gabbaï’s group, and their ability to perform single-electron oxidation of bromide and iodide anions was demonstrated [98–99]. In Ichikawa’s phenolation reaction, the bis-tritylium dication **116^++^** acts as a two-electron oxidant or a PCET agent for phenol, simultaneously activating both reaction components and, in the process, elegantly forming a C–O bonded product. Such generation of two distinct transient radical and cation-radical intermediates, **118** and **119**, at similar rates is crucial for the reaction but is challenging with other SET oxidants. Coupling of these transient open-shell species would provide a sulfenium **120**, which rearranges by a 1,2-shift into a dienone-type intermediate **121**, eventually releasing the thiol radical to furnish the addition–elimination-type product. In principle, the one-electron oxidation of the substrate by bis-tritylium dication can be hypothesized to formally proceed via an unusual structure, **116^+•^**, with a one-electron σ-bond. Recently, the plausibility of such bonding was reported by Shimajiri and Ishigaki [100].

**Scheme 25:**
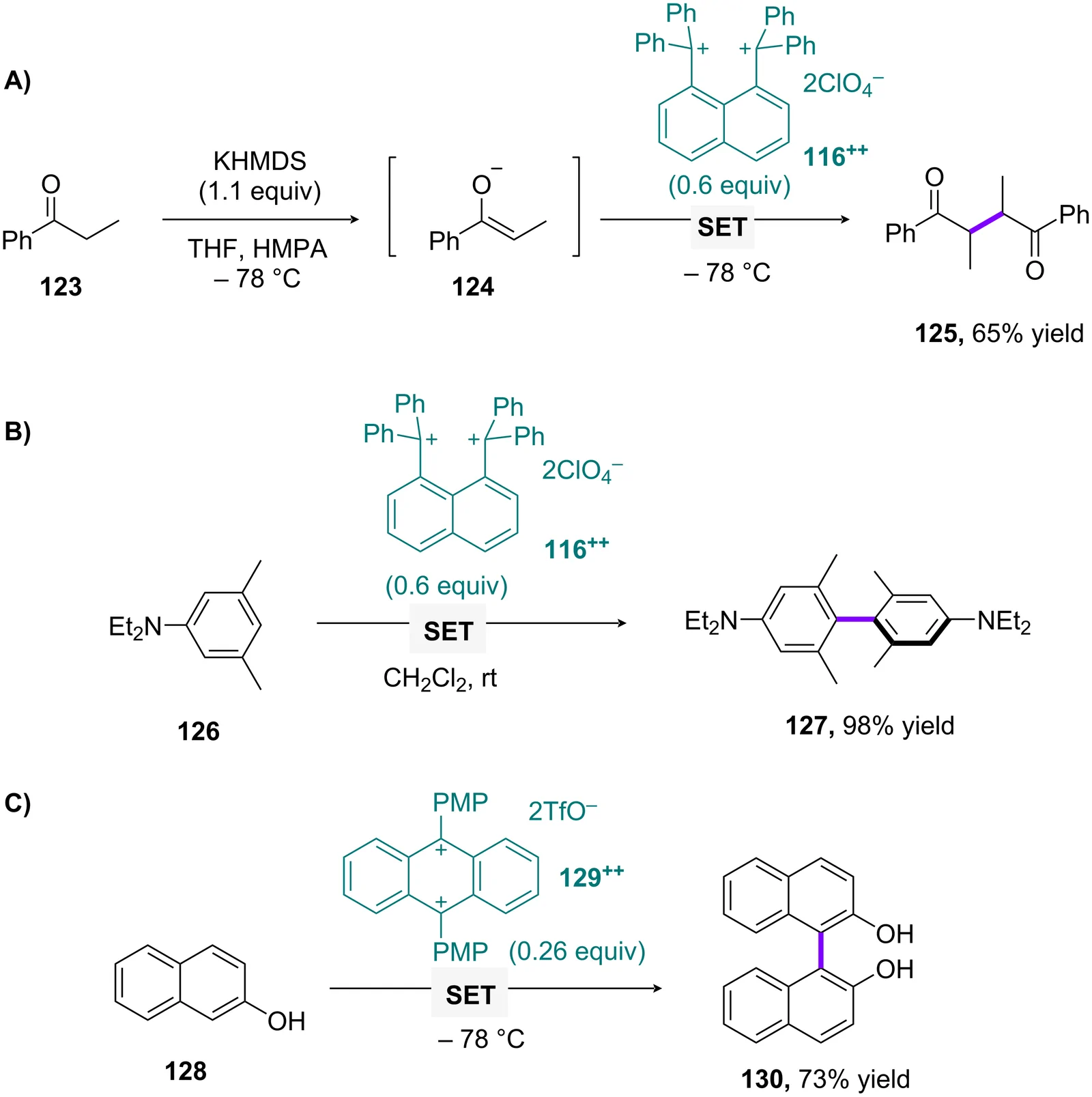
A) Oxidative homocoupling of ketones via SET-oxidation of enolate [101]. B) Oxidative homocoupling of anilines [102]. C) Oxidative homocoupling of naphthol [103].

Ichikawa’s group also reported the use of the same bis-tritylium oxidant in homocoupling reactions of enolates **124** and anilines **126** (Scheme 25A and B) [101–102]. Further studies in this area provided a second-generation dication **129^++^**, which was used in the oxidative dimerization of naphthol **128** (Scheme 25C) [103].

**Scheme 26:**
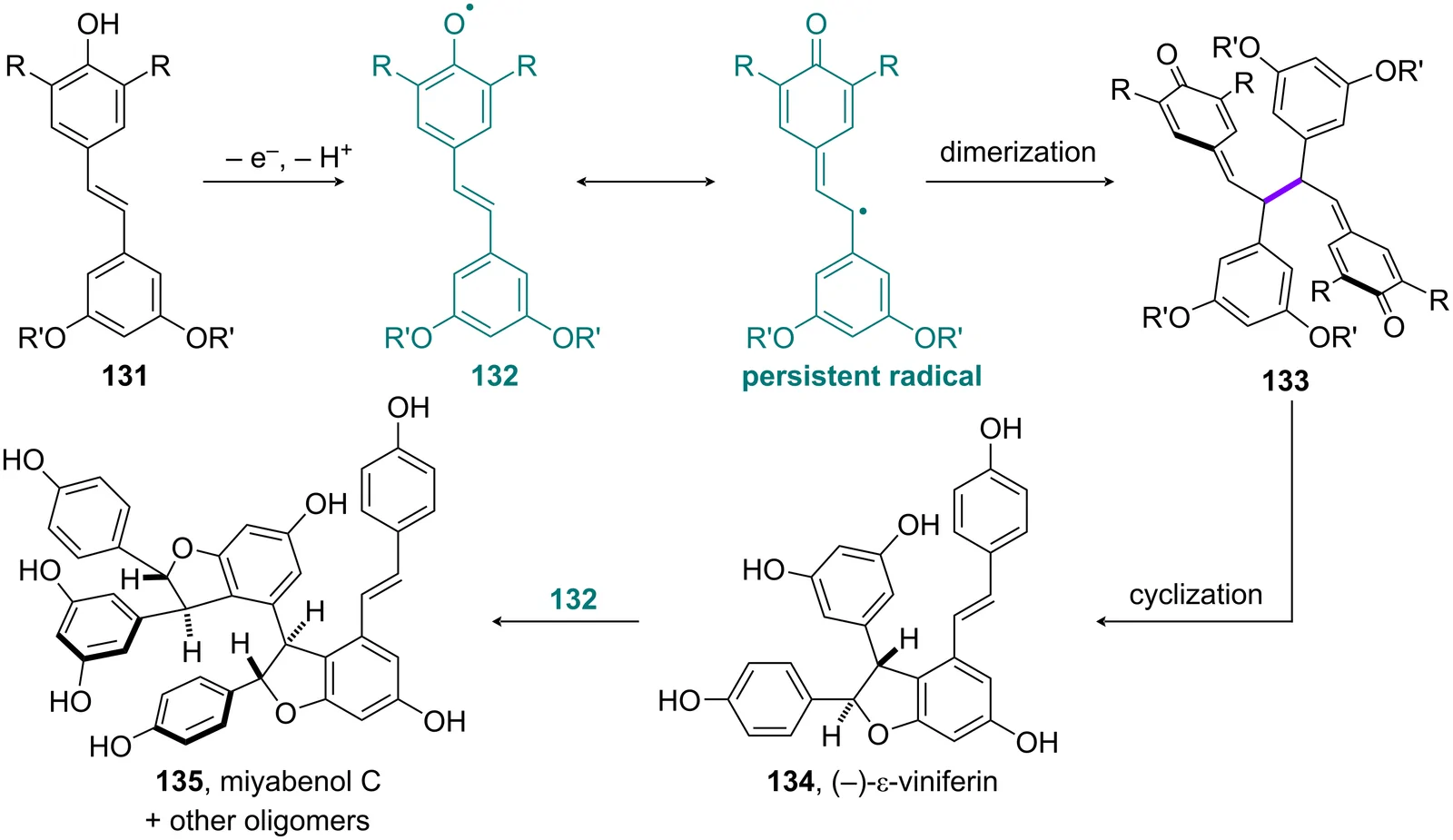
Oxidative dimerization of resveratrol through persistent stilbene radical [106–107].

The stability of phenoxy radicals has long been recognized [104]. However, their interesting reactivity was first noted by Becker in 1969, when he discovered that DDQ oxidation of hydroxystilbenes yielded dimerization products [105]. This approach was used by many groups in studies of natural product synthesis, as many naturally occurring phenolic compounds are thought to originate biosynthetically via resveratrol dimerizations, followed by diverse cyclization patterns. Such persistent radicals **132** could be generated from stilbenes through SET-oxidation of the corresponding phenol in solution, giving a reversible, stereoconvergent radical–dimer equilibrium. Leveraging this behavior, the Stevenson group synthesized a myriad of natural products (Scheme 26) [106]. Later, the Stevenson group also achieved controlled assembly of tetramer derivatives from such stilbenes [107] and published a comprehensive review on this topic [108].

**Scheme 27:**
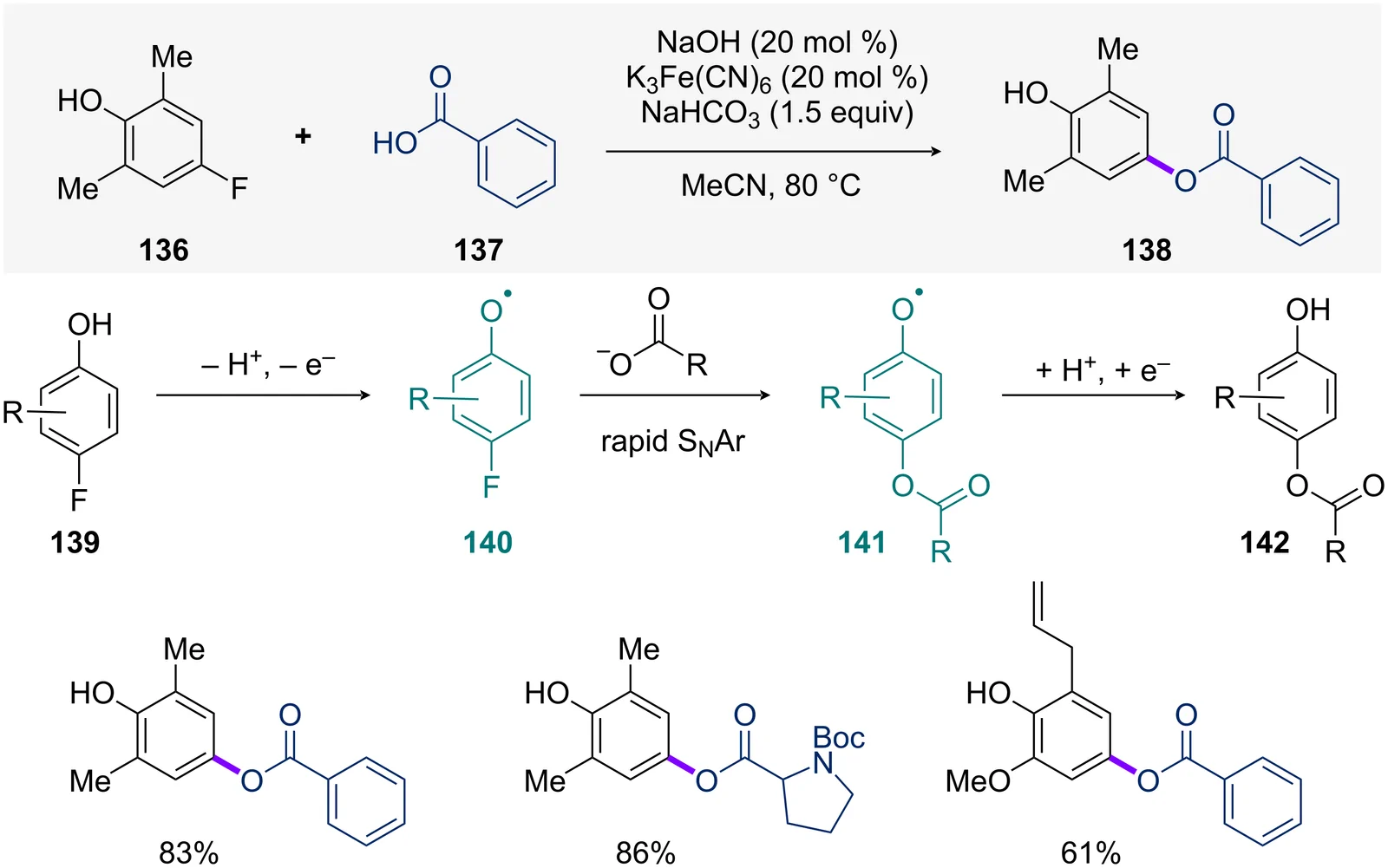
Persistent phenoxyl radical enabled accelerated S_N_Ar [109].

In 2022, an unexpected discovery from the Knowles group revealed that aryloxyl radicals (**140**) are particularly efficient at undergoing S_N_Ar substitution at the *para*-position (Scheme 27) [109]. It should be noted that the persistence of aryloxyl radicals allows both their generation under mild conditions and sufficient lifetime to undergo the substitution reaction by a weak nucleophile such as benzoate.

## Outlook

**Scheme 28:**
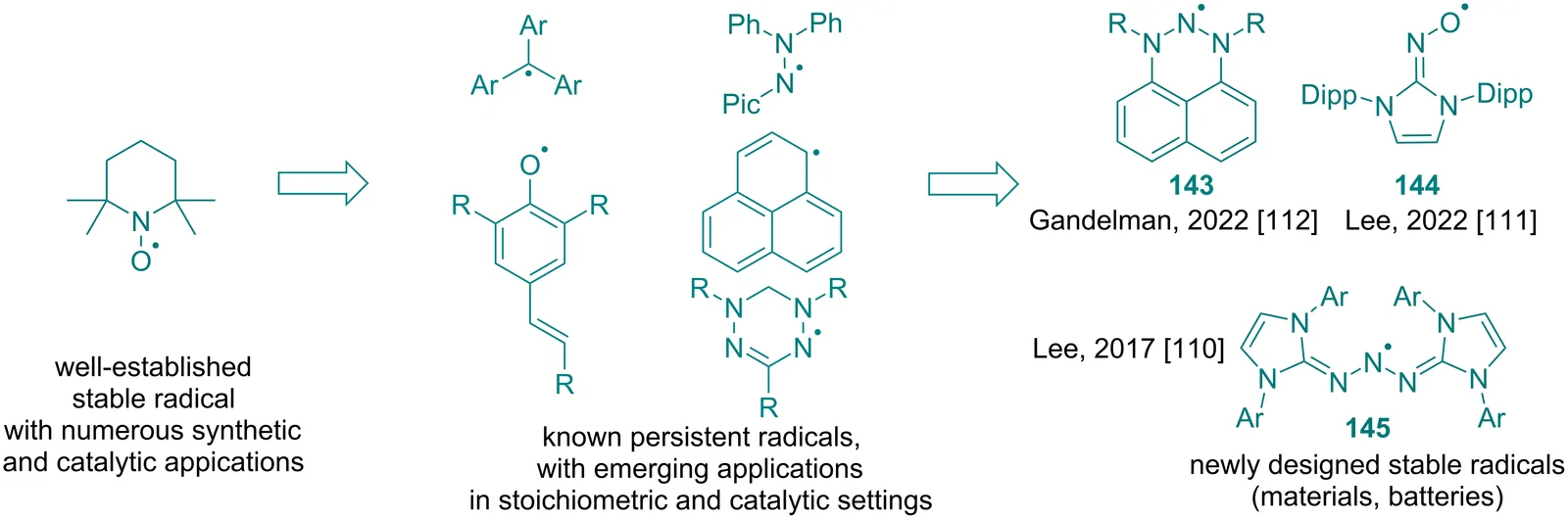
Evolution of uses of persistent radicals in organic synthesis and catalysis beyond nitroxides and TEMPO [110–112].

As the field of stable radicals continues to grow, many types of persistent radicals are being designed. Although the initial motivation is often in materials chemistry, battery research, and redox-responsive functional materials, their application in catalysis can follow in due course [110–113]. Recent reports of novel classes of stable heteroatom-centered persistent radicals, including those based on triazenyl systems and stabilized by N-heterocyclic carbenes, among others, are expected to enable exciting catalytic applications (Scheme 28).

**Scheme 29:**
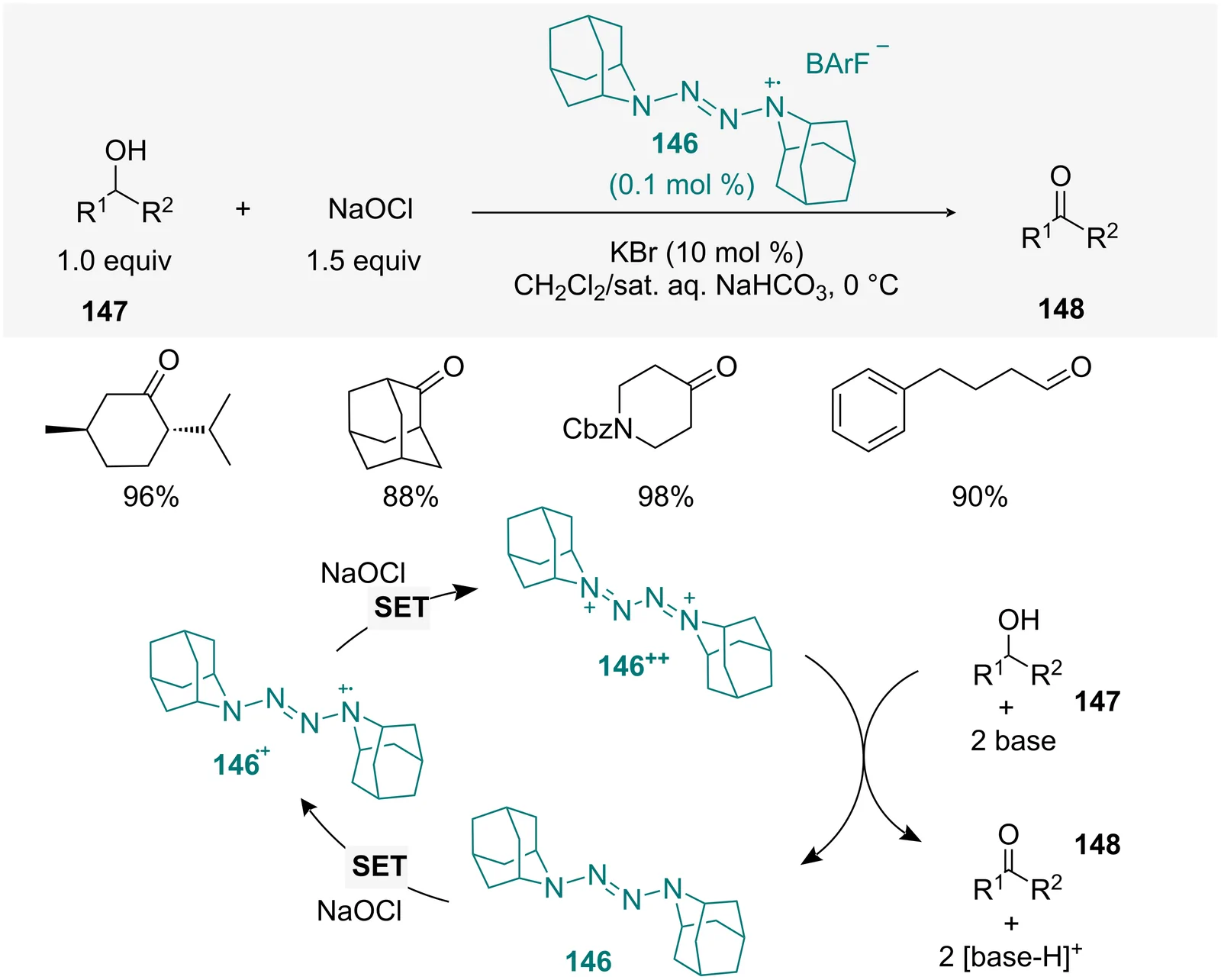
Tetrazene radical-cation-catalyzed oxidation of alcohols [114].

One of the most recent additions in this field is from a team led by Sasano and Iwabuchi, who reported an unusual tetrazene radical cation salt, **146^+•^**, capable of catalyzing the oxidation of sterically challenging alcohols, either electrochemically or with NaOCl as the terminal oxidant (Scheme 29) [114]. Impressively, the radical cation salt could be purified by silica gel column chromatography and is highly air-stable.

## Conclusion

In summary, the redox versatility of organic persistent radicals can be harnessed to drive a wide range of single-electron-transfer (SET) transformations. By exploiting their redox amphoterism, diverse mechanistic manifolds can be accessed involving the three stable redox states of persistent radicals. In addition to classical approaches to PRE-governed processes, emerging frustrated radical-pair systems offer new routes to PRE-controlled reactivity.

More recent advances demonstrate that the synthetic and catalytic potential of persistent radicals extends far beyond established nitroxide systems such as TEMPO. Importantly, alternative persistent radicals should not be viewed simply as nitroxide surrogates; rather, their distinct electronic structures and reactivity profiles enable transformations that are challenging or inaccessible to nitroxides. In particular, electron-rich persistent radicals exhibit enhanced redox activity, facilitating substrate activation and reaction manifolds beyond the reach of conventional nitroxide chemistry. Nitrogen-centered radicals are particularly notable for their ability to mediate ground-state single-electron shuttle catalysis, mimicking key features of transition-metal and photoredox catalysts while offering complementary and potentially more sustainable approaches to radical generation.

Continued development of persistent radical platforms, guided by systematic tuning of their electronic properties, is expected to unlock increasingly challenging substrate classes and broaden the scope of ground-state redox organocatalysis. As the range of accessible persistent radical scaffolds continues to expand, their diverse redox properties are likely to enable new modes of substrate activation and catalysis.

## Data Availability

Data sharing is not applicable as no new data was generated or analyzed in this study.
